# Supplementary material for: Habitat suitability of cetaceans in the Gulf of Mexico using an ecological niche modeling approach
Source: PeerJ. 2021 Mar 17;9:e10834. doi: 10.7717/peerj.10834 (PMC7980700; doi:10.7717/peerj.10834)

Supplemental Information S2. Maps of the sightings records for each species: total records and used records after the spatial filtering, and partitioned records for evaluation and training the models.

Habitat suitability of cetaceans in the Gulf of Mexico using an ecological niche modeling approach.

M. Rafael Ramírez-León[1], María C. García-Aguilar[2], Alfonsina E. Romo-Curiel[2], Zurisaday Ramírez-Mendoza[2], Arturo Fajardo-Yamamoto[2], Oscar Sosa Nishizaki[2]

[1]Posgrado en Ecología Marina, Centro de Investigación Científica y de Educación Superior de Ensenada, Baja California (CICESE), Carretera Ensenada-Tijuana Nº 3918, Ensenada, Baja California, 22860, Mexico.

[2]Departamento de Oceanología Biológica, CICESE, Carretera Ensenada-Tijuana Nº 3918, Ensenada, Baja California, 22860, Mexico.

Corresponding Author:

María C. García-Aguilar

Carretera Ensenada-Tijuana Nº 3918, Ensenada, Baja California, 22860, Mexico.

Email address: gaguilar@cicese.mx

Figure S1. (A) Total records found and used records after the spatial filtering for the sperm whale. (B) Partitioned records for evaluation and training the models.

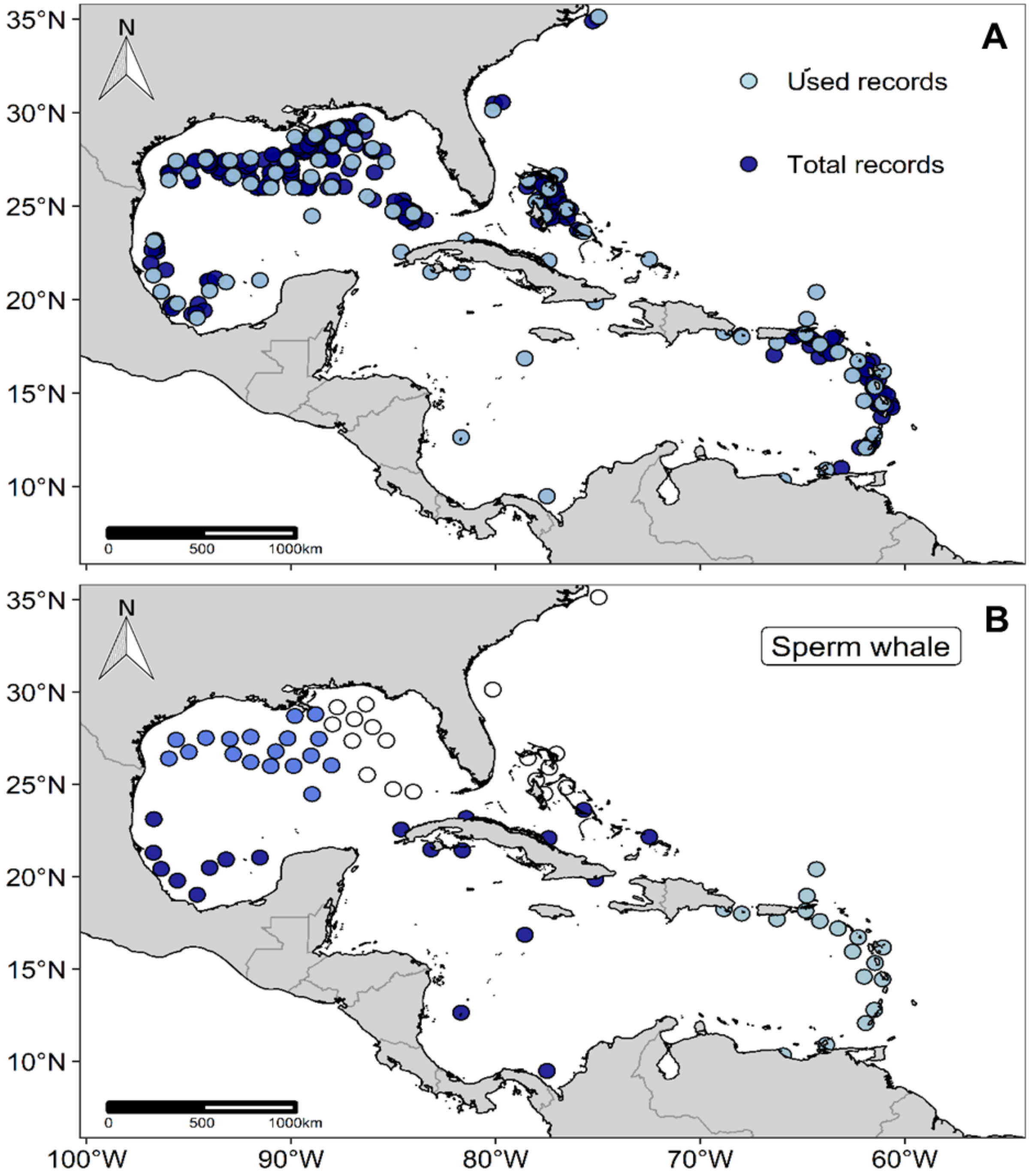

Figure S2. (A) Total records found and used records after the spatial filtering for the dwarf sperm whale. (B) Partitioned records for evaluation and training the models.

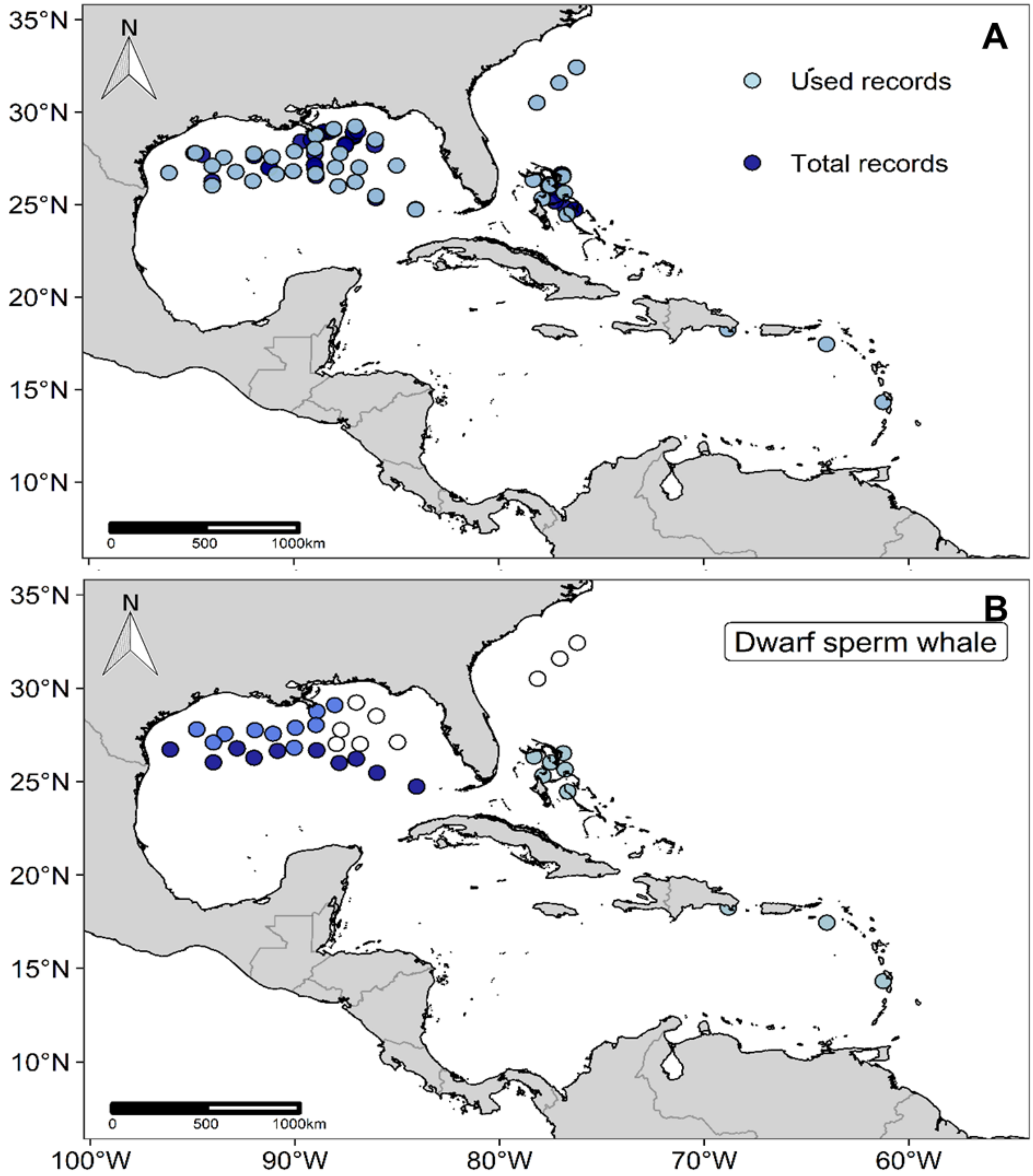

Figure S3. (A) Total records found and used records after the spatial filtering for the Cuvier's beaked whale. (B) Partitioned records for evaluation and training the models.

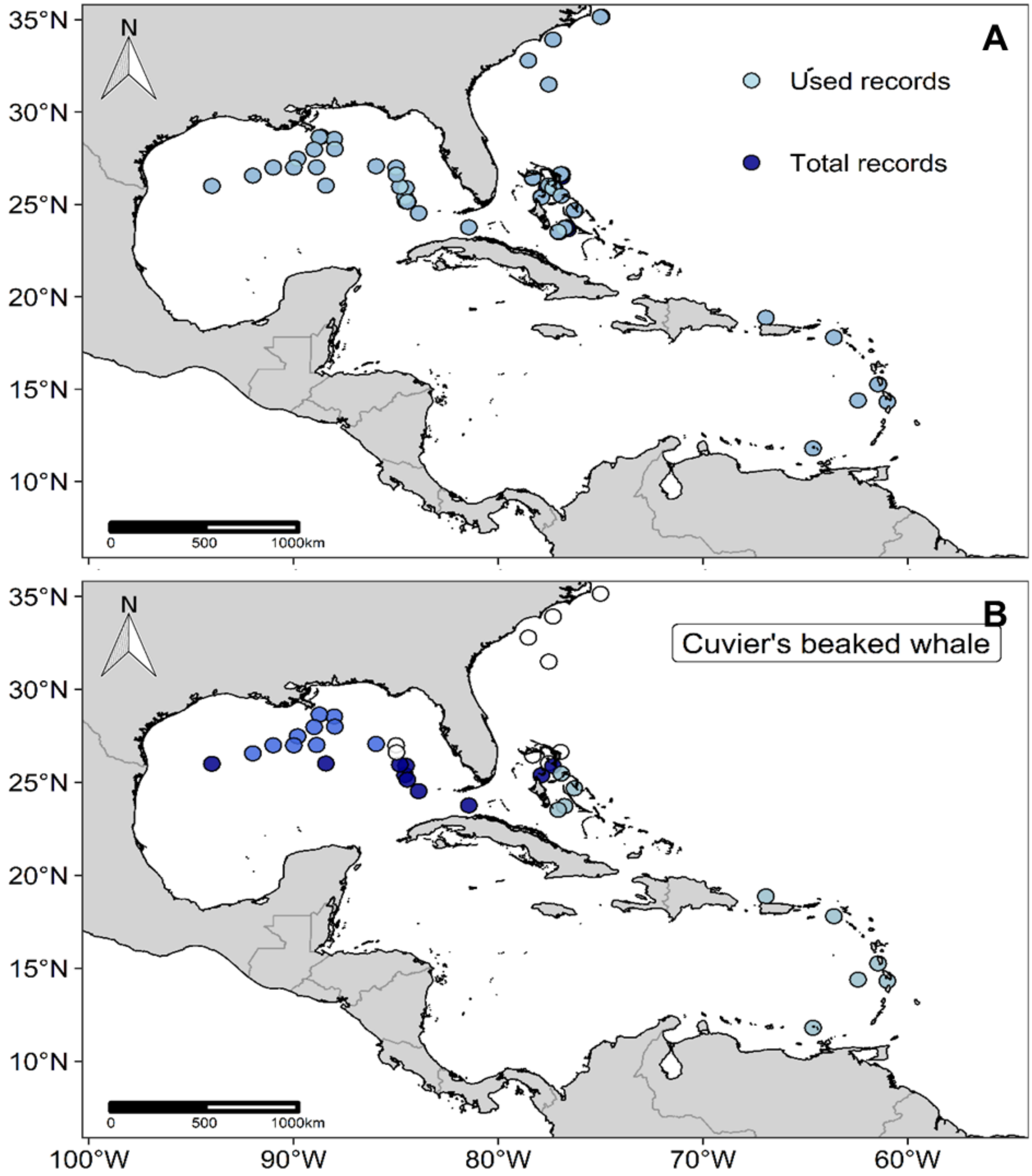

Figure S4. (A) Total records found and used records after the spatial filtering for the short-finned pilot whale. (B) Partitioned records for evaluation and training the models.

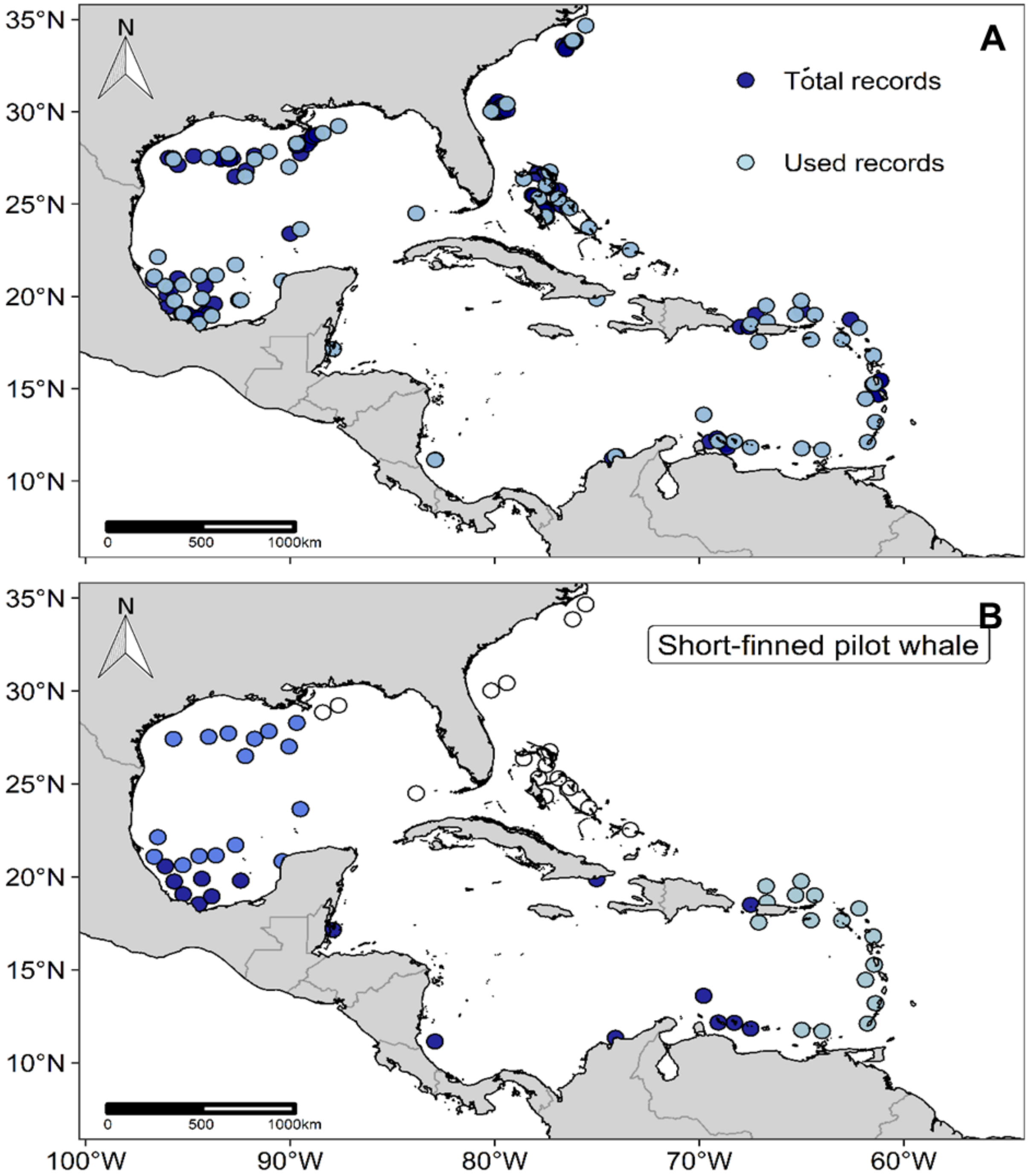

Figure S5. (A) Total records found and used records after the spatial filtering for the rough-toothed dolphin. (B) Partitioned records for evaluation and training the models.

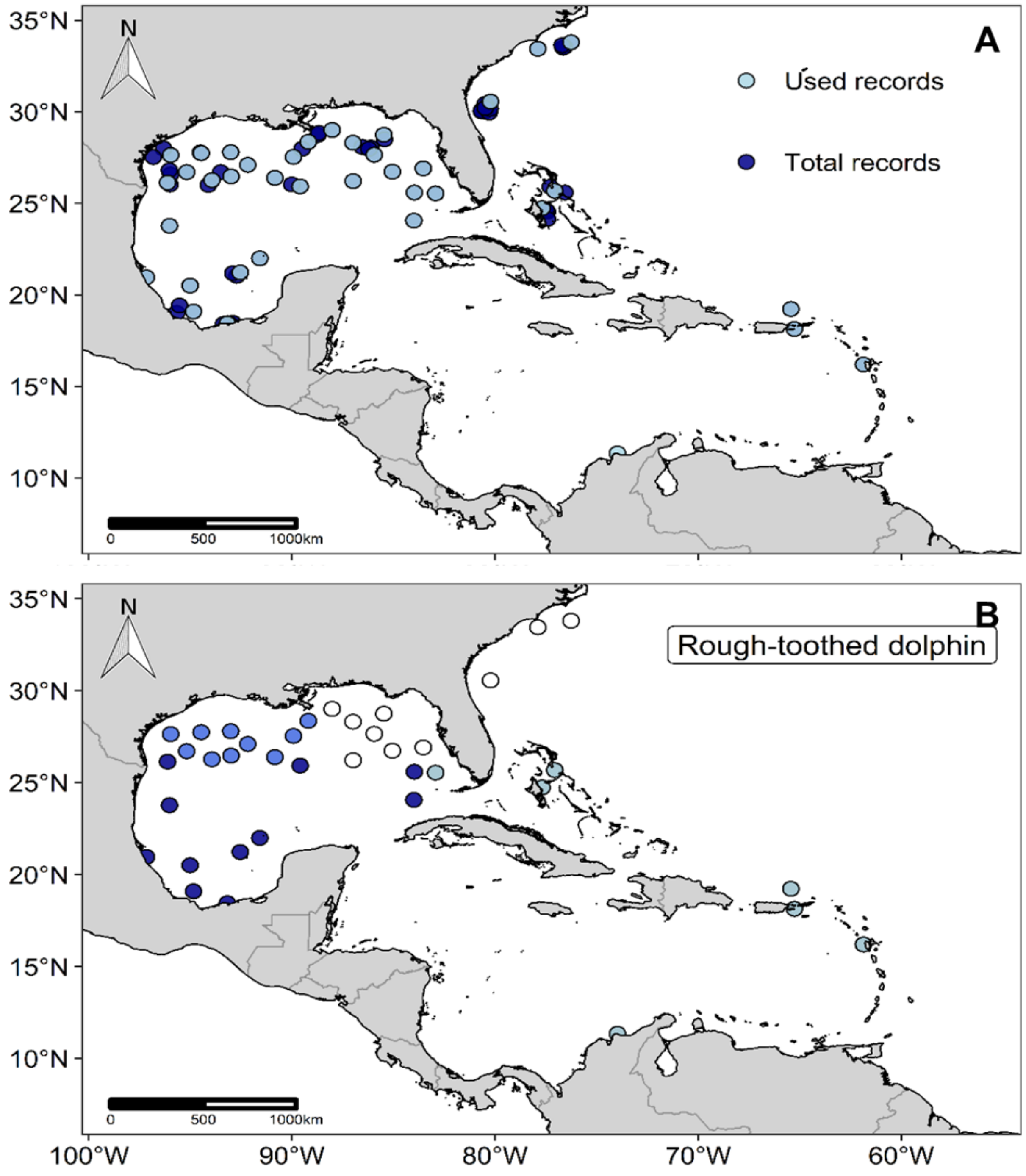

Figure S6. (A) Total sightings found and used sightings after the spatial filtering for the Risso's dolphin. (B) Partitioned records for evaluation and training the models.

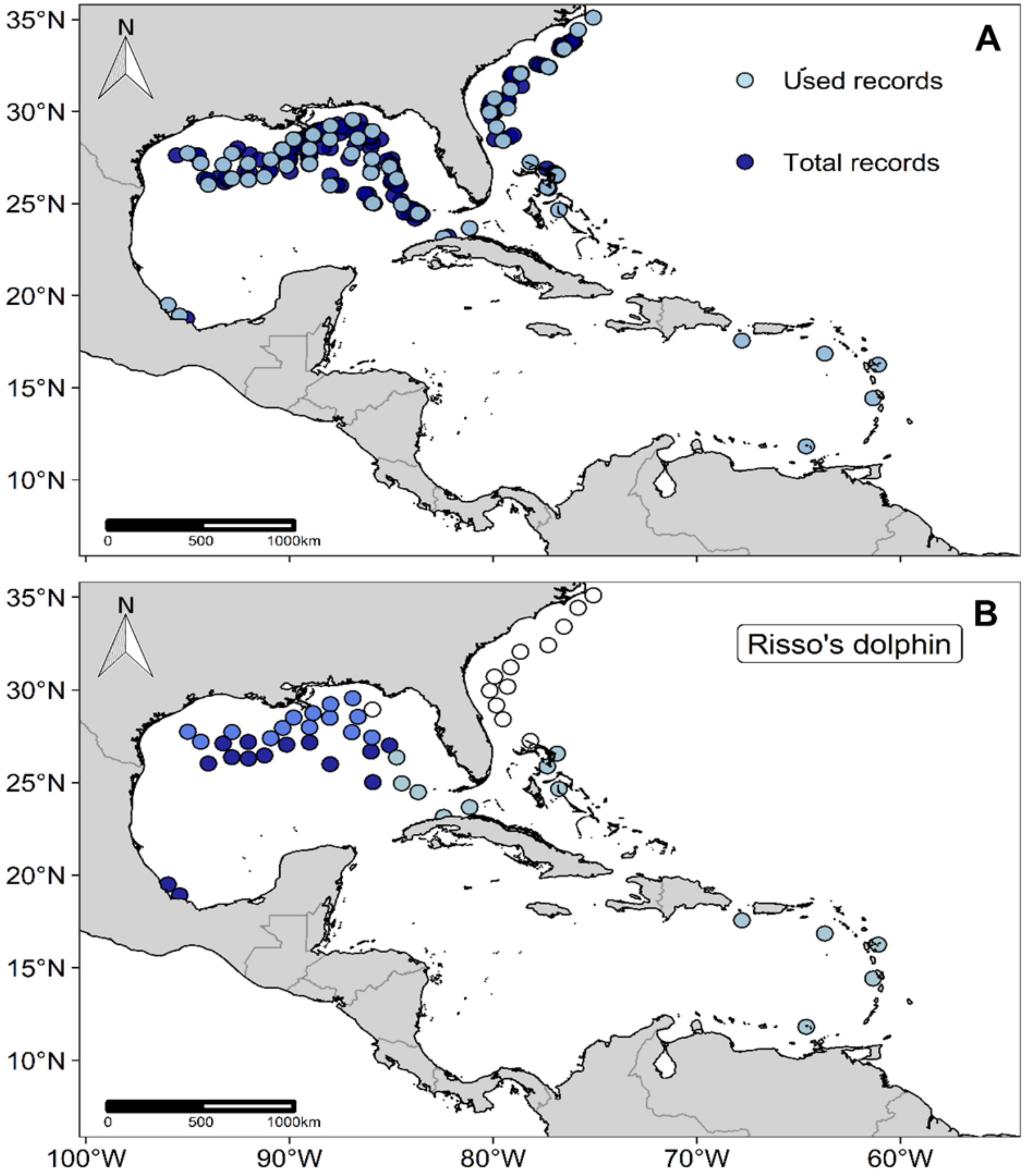

Figure S7. (A) Total records found and used records after the spatial filtering for the Atlantic spotted dolphin. (B) Partitioned records for evaluation and training the models.

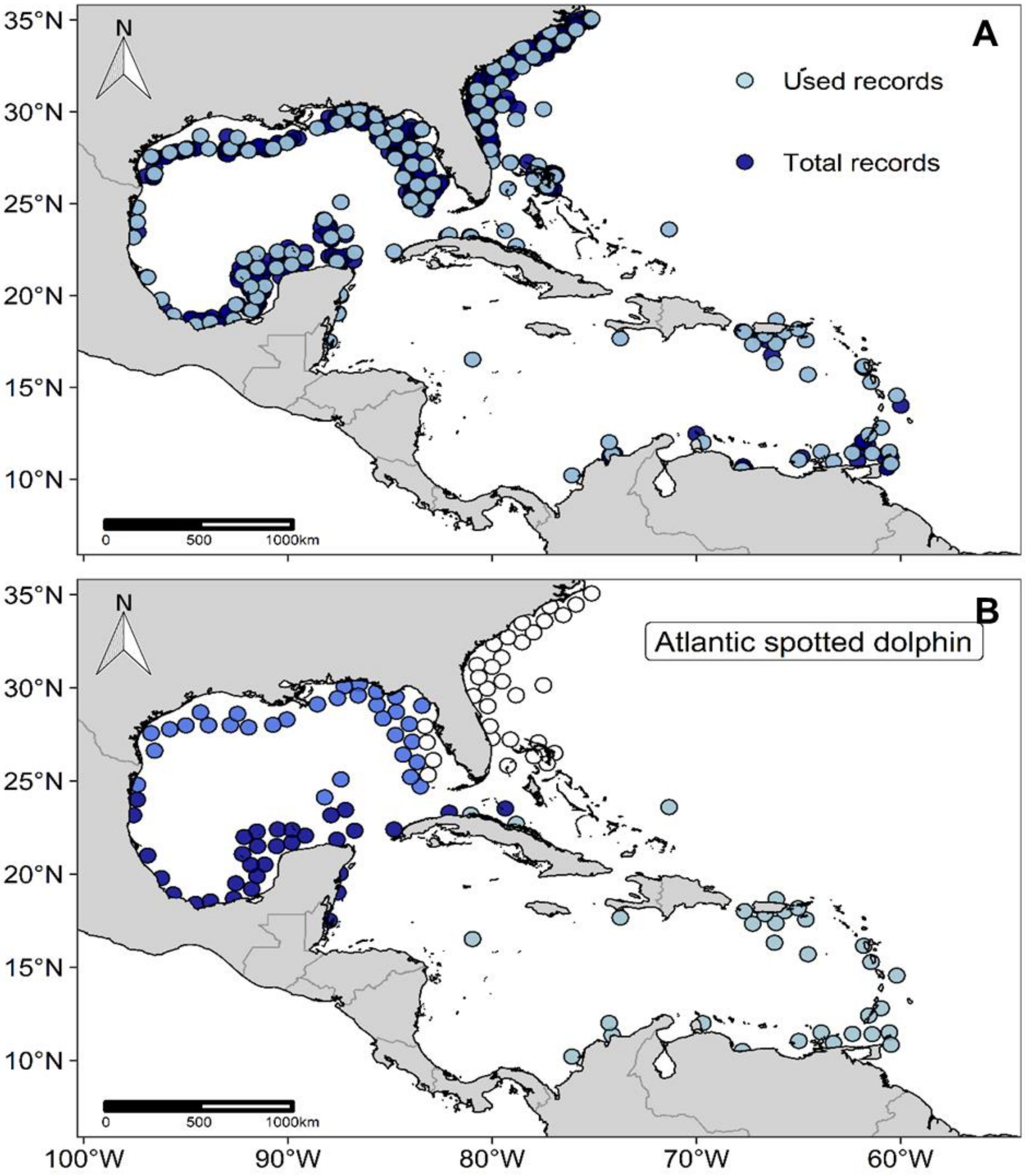

Figure S8. (A) Total records found and used records after the spatial filtering for the pantropical spotted dolphin. (B) Partitioned records for evaluation and training the models.

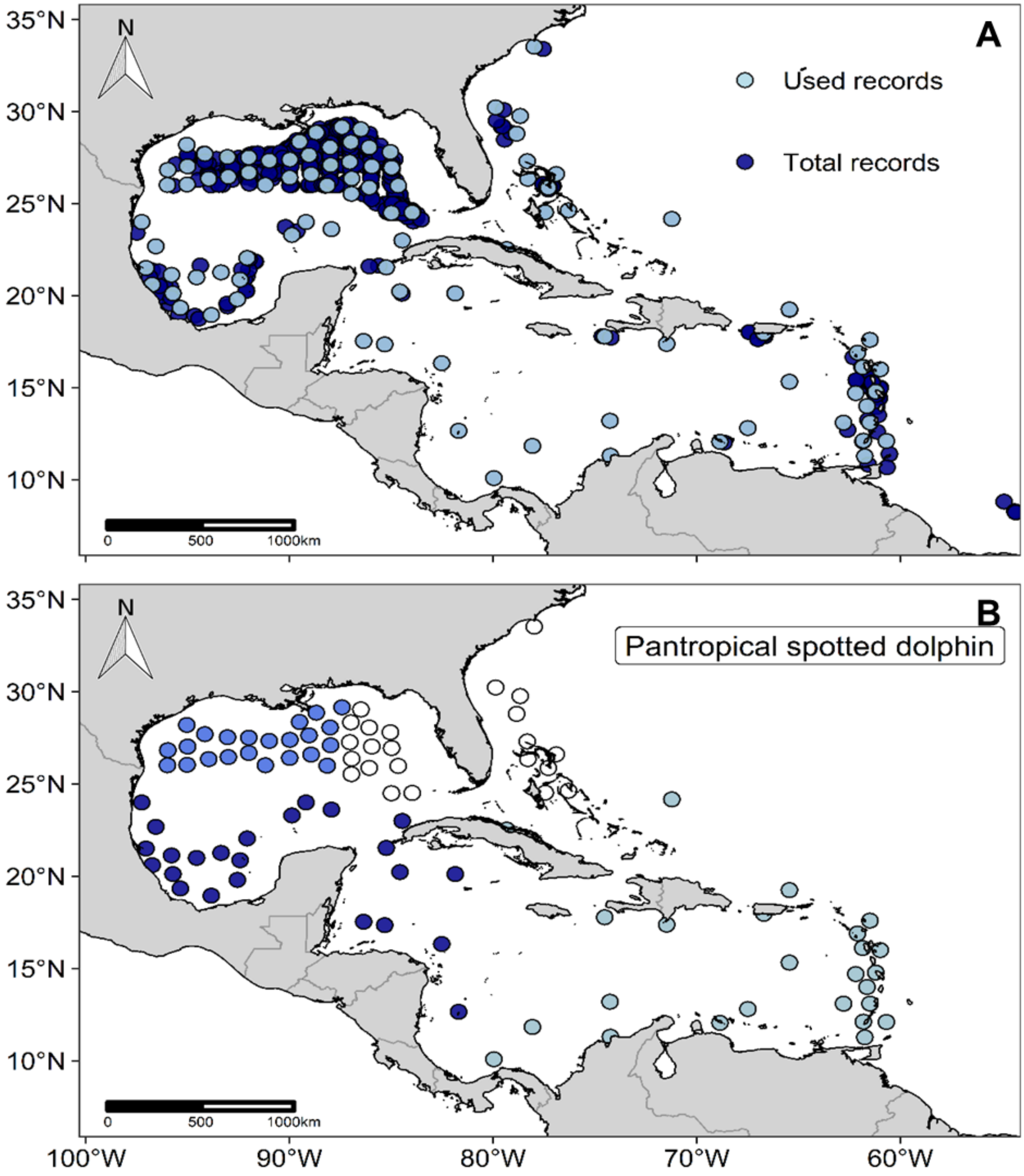

Figure S9. (A) Total records found and used records after the spatial filtering for the striped dolphin. (B) Partitioned records for evaluation and training the models.

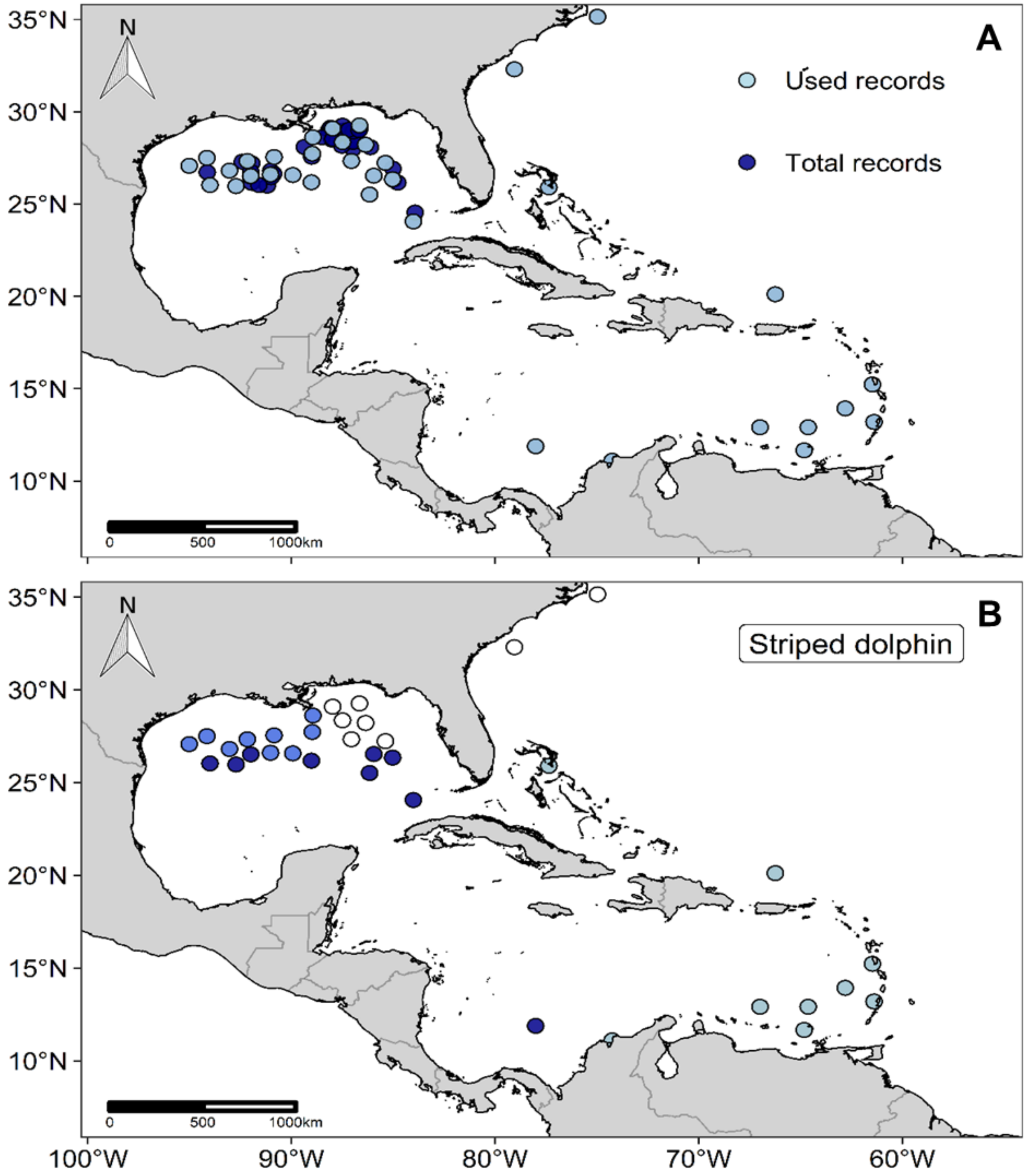

Figure S10. (A) Total records found and used records after the spatial filtering for the spinner dolphin. (B) Partitioned records for evaluation and training the models.

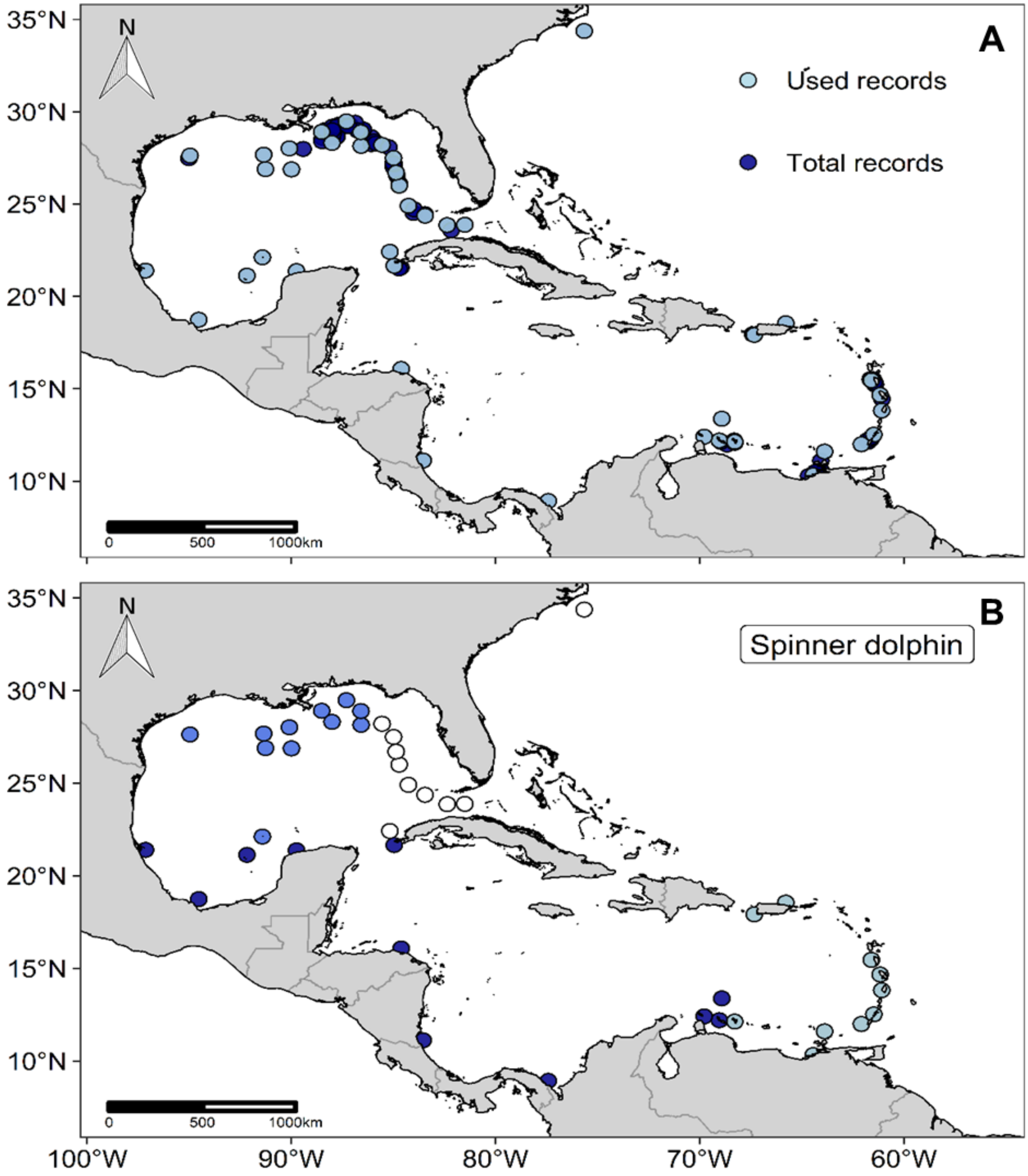

Figure S11. (A) Total records found and used records after the spatial filtering for the Clymene dolphin. (B) Partitioned records for evaluation and training the models.

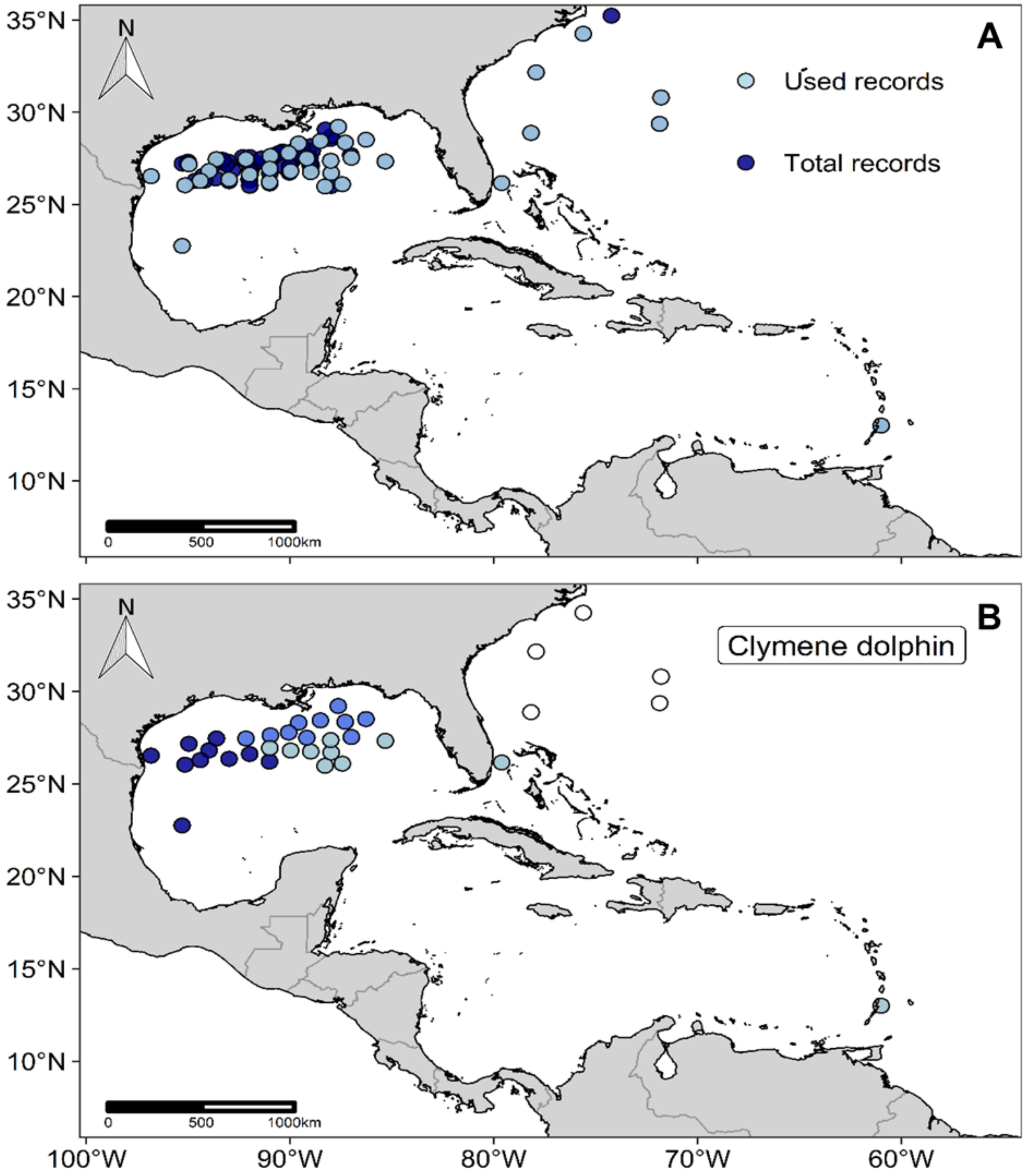

Figure S12. (A) Total records found and used records after the spatial filtering for the bottlenose dolphin. (B) Partitioned records for evaluation and training the models.

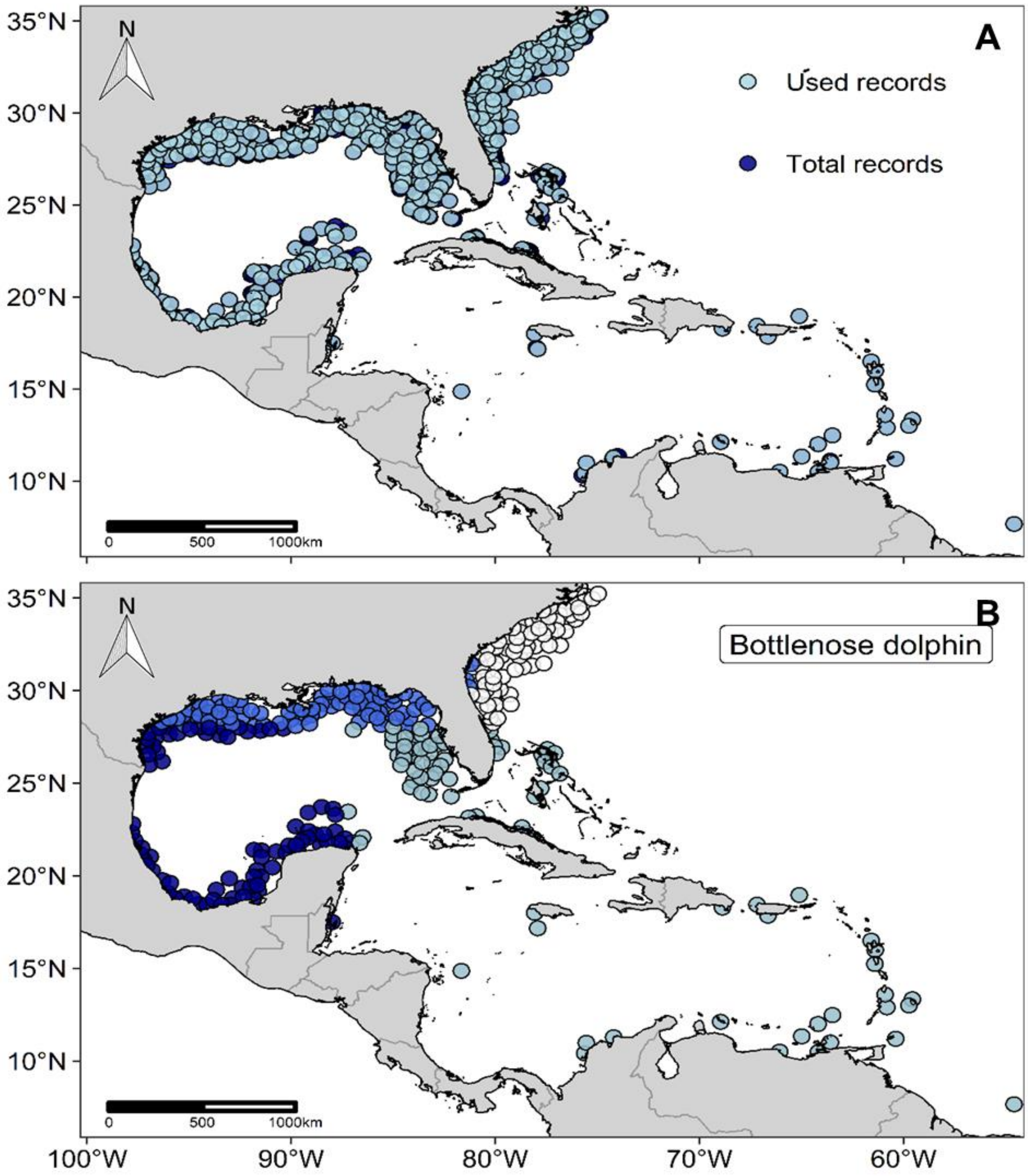

Supplement: Supplemental Information 2 — Figure S1.(A) Total records found and used records after the spatial filtering for the sperm whale. (B) Partitioned records for evaluation and training the models. Figure S2. (A) Total records found and used records after the spatial filtering for the dwarf sperm whale. (B) Partitioned records for evaluation and training the models. Figure S3. (A) Total records found and used records after the spatial filtering for the Cuvier’s beaked whale. (B) Partitioned records for evaluation and training the models. Figure S4. (A) Total records found and used records after the spatial filtering for the short-finned pilot whale. (B) Partitioned records for evaluation and training the models. Figure S5. (A) Total records found and used records after the spatial filtering for the rough-toothed dolphin. (B) Partitioned records for evaluation and training the models. Figure S6. (A) Total sightings found and used sightings after the spatial filtering for the Risso’s dolphin. (B) Partitioned records for evaluation and training the models. Figure S7. (A) Total records found and used records after the spatial filtering for the Atlantic spotted dolphin. (B) Partitioned records for evaluation and training the models. Figure S8. (A) Total records found and used records after the spatial filtering for the pantropical spotted dolphin. (B) Partitioned records for evaluation and training the models. Figure S9. (A) Total records found and used records after the spatial filtering for the striped dolphin. (B) Partitioned records for evaluation and training the models. Figure S10. (A) Total records found and used records after the spatial filtering for the spinner dolphin. (B) Partitioned records for evaluation and training the models. Figure S11. (A) Total records found and used records after the spatial filtering for the Clymene dolphin. (B) Partitioned records for evaluation and training the models. Figure S12. (A) Total records found and used records after the spatial filtering for the bottlen [file peerj-09-10834-s002.pdf]
