# Supplementary material for: Habitat suitability of cetaceans in the Gulf of Mexico using an ecological niche modeling approach
Source: PeerJ. 2021 Mar 17;9:e10834. doi: 10.7717/peerj.10834 (PMC7980700; doi:10.7717/peerj.10834)
Supplement: Supplemental Information 3 — Table S1.Sperm whale model. Table S2. Dwarf sperm whale model. Table S3. Cuvier’s beaked whale model. Table S4. Short-finned pilot whale model. Table S5. Rough-toothed dolphin model. Table S6. Risso’s dolphin model. Table S7. Atlantic spotted dolphin model. Table S8. Pantropical spotted dolphin model. Table S9. Striped dolphin model. Table S10. Spinner dolphin model. Table S11. Clymene dolphin model. Table S12. Bottlenose dolphin model. [file peerj-09-10834-s003.docx]

Supplemental Information S3. Summary of the principal component analysis (PCA) of the environmental predictors of each model

**Habitat suitability of cetaceans in the Gulf of Mexico using an ecological niche modeling approach.**

M. Rafael Ramírez-León^1^, María C. García-Aguilar^2^, Alfonsina E. Romo-Curiel^2^, Zurisaday Ramírez-Mendoza^2^, Arturo Fajardo-Yamamoto^2^, Oscar Sosa Nishizaki^2^

^1^Posgrado en Ecología Marina, Centro de Investigación Científica y de Educación Superior de Ensenada, Baja California (CICESE), Carretera Ensenada-Tijuana Nº 3918, Ensenada, Baja California, 22860, Mexico.

^2^Departamento de Oceanología Biológica, CICESE, Carretera Ensenada-Tijuana Nº 3918, Ensenada, Baja California, 22860, Mexico.

Corresponding Author:

María C. García-Aguilar

Carretera Ensenada-Tijuana Nº 3918, Ensenada, Baja California, 22860, Mexico.

Email address: gaguilar@cicese.mx

**Table S1.** Sperm whale model.

|  | **PC1** | **PC2** | **PC3** |
| --- | --- | --- | --- |
| **SS loadings** | 2.89 | 2.38 | 1.82 |
| **Proportion Variance** | 0.32 | 0.26 | 0.20 |
| **Cumulative Variance** | 0.32 | 0.59 | 0.79 |
| **Proportion explained** | 0.41 | 0.34 | 0.26 |
| **Cumulative proportion** | 0.41 | 0.74 | 1.00 |
| **Environmental predictors** |  |  |  |
| SST_m_ | 0.87 | -0.24 | -0.12 |
| SST_min_ | 0.90 | -0.27 | 0.03 |
| SST_max_ | -0.73 | 0.27 | -0.31 |
| Chl-*a*_m_ | -0.23 | 0.93 | 0.08 |
| Chl-*a*_min_ | 0.17 | 0.62 | 0.50 |
| Chl-*a_max_* | -0.20 | 0.93 | 0.07 |
| *D* | -0.24 | 0.09 | 0.91 |
| *S* | 0.67 | 0.17 | 0.03 |
| *D*_200_ | -0.39 | -0.14 | -0.79 |

**Table S2.** Dwarf sperm whale model.

|  | **PC1** | **PC2** | **PC3** |
| --- | --- | --- | --- |
| **SS loadings** | 2.70 | 2.58 | 2.27 |
| **Proportion Variance** | 0.30 | 0.29 | 0.25 |
| **Cumulative Variance** | 0.30 | 0.59 | 0.84 |
| **Proportion explained** | 0.36 | 0.34 | 0.30 |
| **Cumulative proportion** | 0.36 | 0.70 | 1.00 |
| **Environmental predictors** |  |  |  |
| SST_m_ | -0.53 | 0.74 | -0.11 |
| SST_min_ | -0.22 | 0.88 | -0.28 |
| SST_max_ | -0.39 | -0.66 | 0.36 |
| Chl-*a*_m_ | 0.18 | -0.15 | 0.95 |
| Chl-*a*_min_ | 0.7 | -0.12 | 0.45 |
| Chl-*a_max_* | 0.15 | -0.15 | 0.96 |
| *D* | 0.94 | -0.11 | 0.06 |
| *S* | 0.23 | 0.82 | 0.03 |
| *D*_200_ | -0.86 | -0.31 | -0.16 |

**Table S3.** Cuvier’s beaked whale model.

|  | **PC1** | **PC2** | **PC3** |
| --- | --- | --- | --- |
| **SS loadings** | 2.70 | 2.32 | 1.46 |
| **Proportion Variance** | 0.30 | 0.26 | 0.16 |
| **Cumulative Variance** | 0.30 | 0.56 | 0.72 |
| **Proportion explained** | 0.42 | 0.36 | 0.23 |
| **Cumulative proportion** | 0.42 | 0.77 | 1.00 |
| **Environmental predictors** |  |  |  |
| SST_m_ | -0.75 | 0.51 | -0.18 |
| SST_min_ | -0.59 | 0.64 | -0.24 |
| SST_max_ | -0.18 | -0.74 | 0.15 |
| Chl-*a*_m_ | 0.57 | -0.07 | 0.70 |
| Chl-*a*_min_ | 0.81 | 0.19 | 0.10 |
| Chl-*a_max_* | -0.03 | -0.09 | 0.93 |
| *D* | 0.78 | 0.13 | -0.01 |
| *S* | -0.18 | 0.68 | 0.03 |
| *D*_200_ | -0.38 | -0.76 | 0.03 |

**Table S4.** Short-finned pilot whale model.

|  | **PC1** | **PC2** | **PC3** |
| --- | --- | --- | --- |
| **SS loadings** | 2.79 | 2.37 | 1.66 |
| **Proportion Variance** | 0.31 | 0.26 | 0.18 |
| **Cumulative Variance** | 0.31 | 0.57 | 0.76 |
| **Proportion explained** | 0.41 | 0.35 | 0.24 |
| **Cumulative proportion** | 0.41 | 0.76 | 1.00 |
| **Environmental predictors** |  |  |  |
| SST_m_ | 0.90 | -0.10 | -0.04 |
| SST_min_ | 0.95 | -0.03 | -0.06 |
| SST_max_ | -0.75 | 0.05 | 0.09 |
| Chl-*a*_m_ | -0.29 | 0.92 | 0.10 |
| Chl-*a*_min_ | 0.24 | 0.75 | 0.32 |
| Chl-*a_max_* | -0.20 | 0.90 | 0.02 |
| *D* | -0.33 | 0.19 | 0.82 |
| *S* | 0.47 | -0.30 | 0.25 |
| *D*_200_ | -0.14 | -0.10 | -0.89 |

**Table S5.** Rough-toothed dolphin model.

|  | **PC1** | **PC2** | **PC3** |
| --- | --- | --- | --- |
| **SS loadings** | 2.71 | 2.62 | 1.60 |
| **Proportion Variance** | 0.30 | 0.29 | 0.18 |
| **Cumulative Variance** | 0.30 | 0.59 | 0.77 |
| **Proportion explained** | 0.39 | 0.38 | 0.23 |
| **Cumulative proportion** | 0.39 | 0.77 | 1.00 |
| **Environmental predictors** |  |  |  |
| SST_m_ | 0.14 | 0.92 | 0.06 |
| SST_min_ | 0.13 | 0.92 | -0.10 |
| SST_max_ | 0.02 | -0.07 | 0.76 |
| Chl-*a*_m_ | 0.96 | 0.08 | -0.02 |
| Chl-*a*_min_ | 0.75 | -0.46 | -0.31 |
| Chl-*a_max_* | 0.93 | 0.13 | 0.05 |
| *D* | 0.44 | -0.64 | -0.17 |
| *S* | -0.30 | 0.52 | -0.45 |
| *D*_200_ | -0.21 | 0.13 | 0.82 |

**Table S6.** Risso’s dolphin model.

|  | **PC1** | **PC2** | **PC3** |
| --- | --- | --- | --- |
| **SS loadings** | 2.36 | 2.30 | 2.23 |
| **Proportion Variance** | 0.26 | 0.26 | 0.25 |
| **Cumulative Variance** | 0.26 | 0.52 | 0.77 |
| **Proportion explained** | 0.34 | 0.33 | 0.32 |
| **Cumulative proportion** | 0.34 | 0.68 | 1.00 |
| **Environmental predictors** |  |  |  |
| SST_m_ | -0.22 | 0.85 | -0.25 |
| SST_min_ | -0.07 | 0.92 | -0.26 |
| SST_max_ | -0.44 | -0.49 | 0.33 |
| Chl-*a*_m_ | 0.19 | -0.18 | 0.93 |
| Chl-*a*_min_ | 0.65 | 0.03 | 0.52 |
| Chl-*a_max_* | 0.04 | -0.20 | 0.90 |
| *D* | 0.89 | -0.15 | 0.03 |
| *S* | 0.29 | 0.60 | 0.09 |
| *D*_200_ | -0.89 | -0.17 | -0.15 |

**Table S7.** Atlantic spotted dolphin model.

|  | **PC1** | **PC2** | **PC3** |
| --- | --- | --- | --- |
| **SS loadings** | 2.64 | 2.15 | 1.42 |
| **Proportion Variance** | 0.29 | 0.24 | 0.16 |
| **Cumulative Variance** | 0.29 | 0.53 | 0.69 |
| **Proportion explained** | 0.43 | 0.35 | 0.23 |
| **Cumulative proportion** | 0.43 | 0.77 | 1.00 |
| **Environmental predictors** |  |  |  |
| SST_m_ | 0.83 | -0.15 | 0.32 |
| SST_min_ | 0.88 | -0.17 | 0.23 |
| SST_max_ | -0.49 | 0.01 | 0.30 |
| Chl-*a*_m_ | -0.10 | 0.92 | -0.06 |
| Chl-*a*_min_ | -0.17 | 0.70 | -0.47 |
| Chl-*a_max_* | -0.07 | 0.80 | 0.09 |
| *D* | -0.59 | 0.32 | 0.49 |
| *S* | 0.70 | -0.04 | 0.07 |
| *D*_200_ | -0.22 | 0.12 | -0.84 |

**Table S8.** Pantropical spotted dolphin model.

|  | **PC1** | **PC2** | **PC3** |
| --- | --- | --- | --- |
| **SS loadings** | 2.42 | 2.29 | 1.64 |
| **Proportion Variance** | 0.27 | 0.25 | 0.18 |
| **Cumulative Variance** | 0.27 | 0.52 | 0.71 |
| **Proportion explained** | 0.38 | 0.36 | 0.26 |
| **Cumulative proportion** | 0.38 | 0.74 | 1.00 |
| **Environmental predictors** |  |  |  |
| SST_m_ | 0.07 | 0.94 | -0.04 |
| SST_min_ | 0.01 | 0.94 | -0.16 |
| SST_max_ | 0.08 | -0.10 | 0.57 |
| Chl-*a*_m_ | 0.97 | -0.06 | 0.01 |
| Chl-*a*_min_ | 0.82 | 0.02 | 0.06 |
| Chl-*a_max_* | 0.77 | -0.02 | -0.06 |
| *D* | 0.33 | -0.60 | -0.53 |
| *S* | -0.19 | 0.36 | -0.52 |
| *D*_200_ | -0.20 | 0.14 | 0.85 |

**Table S9.** Striped dolphin model.

|  | **PC1** | **PC2** | **PC3** |
| --- | --- | --- | --- |
| **SS loadings** | 3.32 | 2.77 | 1.81 |
| **Proportion Variance** | 0.37 | 0.31 | 0.20 |
| **Cumulative Variance** | 0.37 | 0.68 | 0.88 |
| **Proportion explained** | 0.42 | 0.35 | 0.23 |
| **Cumulative proportion** | 0.42 | 0.77 | 1.00 |
| **Environmental predictors** |  |  |  |
| SST_m_ | 0.35 | 0.88 | -0.05 |
| SST_min_ | 0.21 | 0.94 | 0.12 |
| SST_max_ | 0.18 | -0.68 | -0.53 |
| Chl-*a*_m_ | 0.98 | 0.06 | 0.00 |
| Chl-*a*_min_ | 0.92 | 0.17 | 0.26 |
| Chl-*a_max_* | 0.97 | 0.04 | -0.03 |
| *D* | 0.50 | -0.69 | 0.31 |
| *S* | -0.04 | 0.22 | 0.84 |
| *D*_200_ | -0.35 | 0.30 | -0.80 |

**Table S10.** Spinner dolphin model.

|  | **PC1** | **PC2** | **PC3** |
| --- | --- | --- | --- |
| **SS loadings** | 2.77 | 2.32 | 1.48 |
| **Proportion Variance** | 0.31 | 0.26 | 0.16 |
| **Cumulative Variance** | 0.31 | 0.57 | 0.73 |
| **Proportion explained** | 0.42 | 0.35 | 0.23 |
| **Cumulative proportion** | 0.42 | 0.77 | 1.00 |
| **Environmental predictors** |  |  |  |
| SST_m_ | 0.87 | -0.17 | 0.06 |
| SST_min_ | 0.93 | 0.02 | 0.08 |
| SST_max_ | -0.80 | -0.10 | 0.10 |
| Chl-*a*_m_ | -0.13 | 0.95 | 0.11 |
| Chl-*a*_min_ | 0.21 | 0.83 | 0.06 |
| Chl-*a_max_* | -0.27 | 0.73 | -0.05 |
| *D* | -0.17 | 0.30 | 0.85 |
| *S* | 0.52 | -0.16 | 0.11 |
| *D*_200_ | -0.30 | 0.17 | -0.84 |

**Table S11.** Clymene dolphin model.

|  | **PC1** | **PC2** | **PC3** |
| --- | --- | --- | --- |
| **SS loadings** | 2.98 | 2.72 | 1.46 |
| **Proportion Variance** | 0.33 | 0.30 | 0.16 |
| **Cumulative Variance** | 0.33 | 0.63 | 0.80 |
| **Proportion explained** | 0.42 | 0.38 | 0.20 |
| **Cumulative proportion** | 0.42 | 0.80 | 1.00 |
| **Environmental predictors** |  |  |  |
| SST_m_ | 0.02 | 0.42 | 0.64 |
| SST_min_ | 0.15 | 0.93 | 0.11 |
| SST_max_ | -0.08 | 0.90 | 0.07 |
| Chl-*a*_m_ | 0.53 | -0.65 | 0.44 |
| Chl-*a*_min_ | 0.74 | -0.55 | -0.15 |
| Chl-*a_max_* | 0.37 | -0.33 | 0.64 |
| *D* | 0.90 | 0.10 | 0.14 |
| *S* | 0.52 | -0.14 | -0.63 |
| *D*_200_ | -0.95 | -0.05 | -0.02 |

**Table S12.** Bottlenose dolphin model.

|  | **PC1** | **PC2** | **PC3** |
| --- | --- | --- | --- |
| **SS loadings** | 2.59 | 2.51 | 1.06 |
| **Proportion Variance** | 0.29 | 0.28 | 0.12 |
| **Cumulative Variance** | 0.29 | 0.57 | 0.68 |
| **Proportion explained** | 0.42 | 0.41 | 0.17 |
| **Cumulative proportion** | 0.42 | 0.83 | 1.00 |
| **Environmental predictors** |  |  |  |
| SST_m_ | 0.86 | -0.26 | 0.20 |
| SST_min_ | 0.82 | -0.37 | 0.08 |
| SST_max_ | -0.04 | 0.19 | 0.89 |
| Chl-*a*_m_ | -0.12 | 0.86 | -0.05 |
| Chl-*a*_min_ | -0.52 | 0.32 | 0.15 |
| Chl-*a_max_* | -0.20 | 0.92 | 0.11 |
| *D* | -0.21 | 0.72 | 0.28 |
| *S* | -0.58 | 0.11 | 0.08 |
| *D*_200_ | 0.69 | 0.22 | -0.34 |
