# Supplementary material for: Habitat suitability of cetaceans in the Gulf of Mexico using an ecological niche modeling approach
Source: PeerJ. 2021 Mar 17;9:e10834. doi: 10.7717/peerj.10834 (PMC7980700; doi:10.7717/peerj.10834)

Supplemental Information S4. Response curves of the environmental predictors that contributed the most to each model

**Habitat suitability of cetaceans in the Gulf of Mexico using an ecological niche modeling approach.**

M. Rafael Ramírez-León[1], María C. García-Aguilar[2], Alfonsina E. Romo-Curiel[2], Zurisaday Ramírez-Mendoza[2], Arturo Fajardo-Yamamoto[2], Oscar Sosa Nishizaki[2]

[1]Posgrado en Ecología Marina, Centro de Investigación Científica y de Educación Superior de Ensenada, Baja California (CICESE), Carretera Ensenada-Tijuana Nº 3918, Ensenada, Baja California, 22860, Mexico.

[2]Departamento de Oceanología Biológica, CICESE, Carretera Ensenada-Tijuana Nº 3918, Ensenada, Baja California, 22860, Mexico.

Corresponding Author:

María C. García-Aguilar

Carretera Ensenada-Tijuana Nº 3918, Ensenada, Baja California, 22860, Mexico.

Email address: gaguilar@cicese.mx

**Figure S1.** Sperm whale model. (A) $SST_{min}$, (B) Chl-$a_{max}$, (C) depth, (D) slope bottom, and (E) distance to the 200-m isobath.

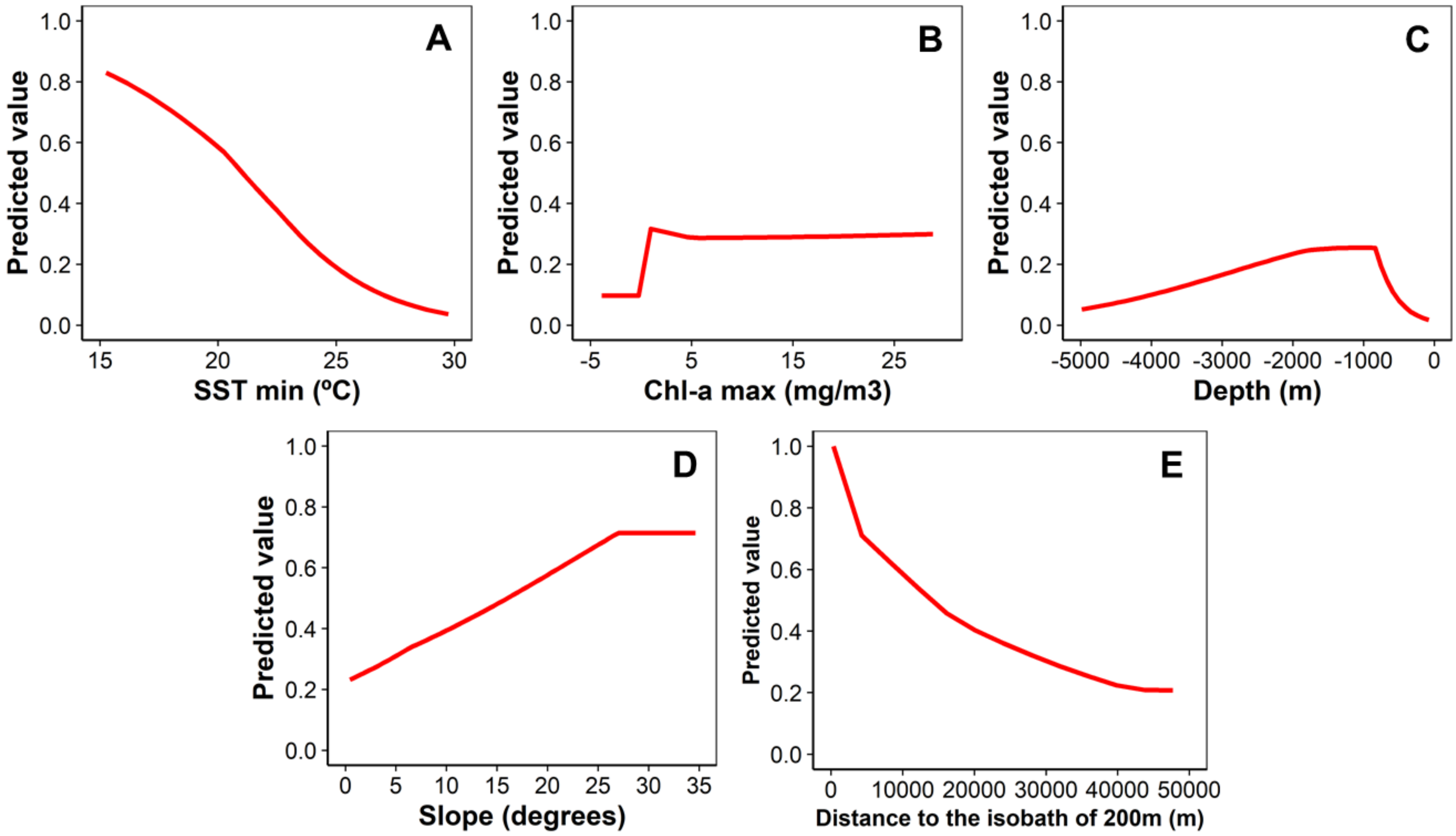

**Figure S2.** Dwarf sperm whale model. (A) $SST_{min}$, (B) $SST_{max}$, (C) Chl-$a_{max}$, (D) depth, and (E) slope bottom.

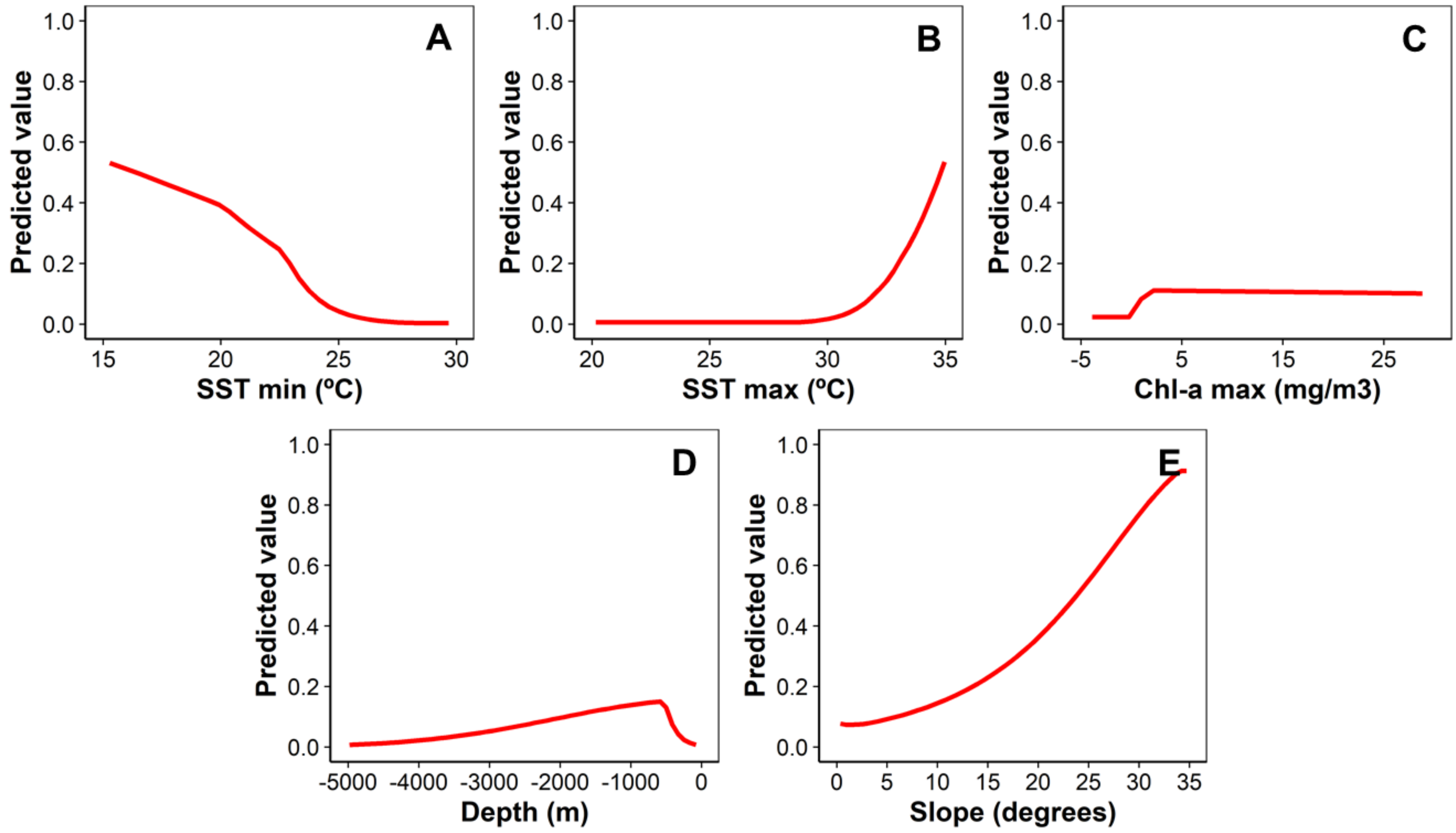

**Figure S3.** Cuvier's beaked whale model. (A) $SST_{min}$, (B) $SST_{max}$, (C) depth, (D) slope bottom, and (E) distance to the 200-m isobath.

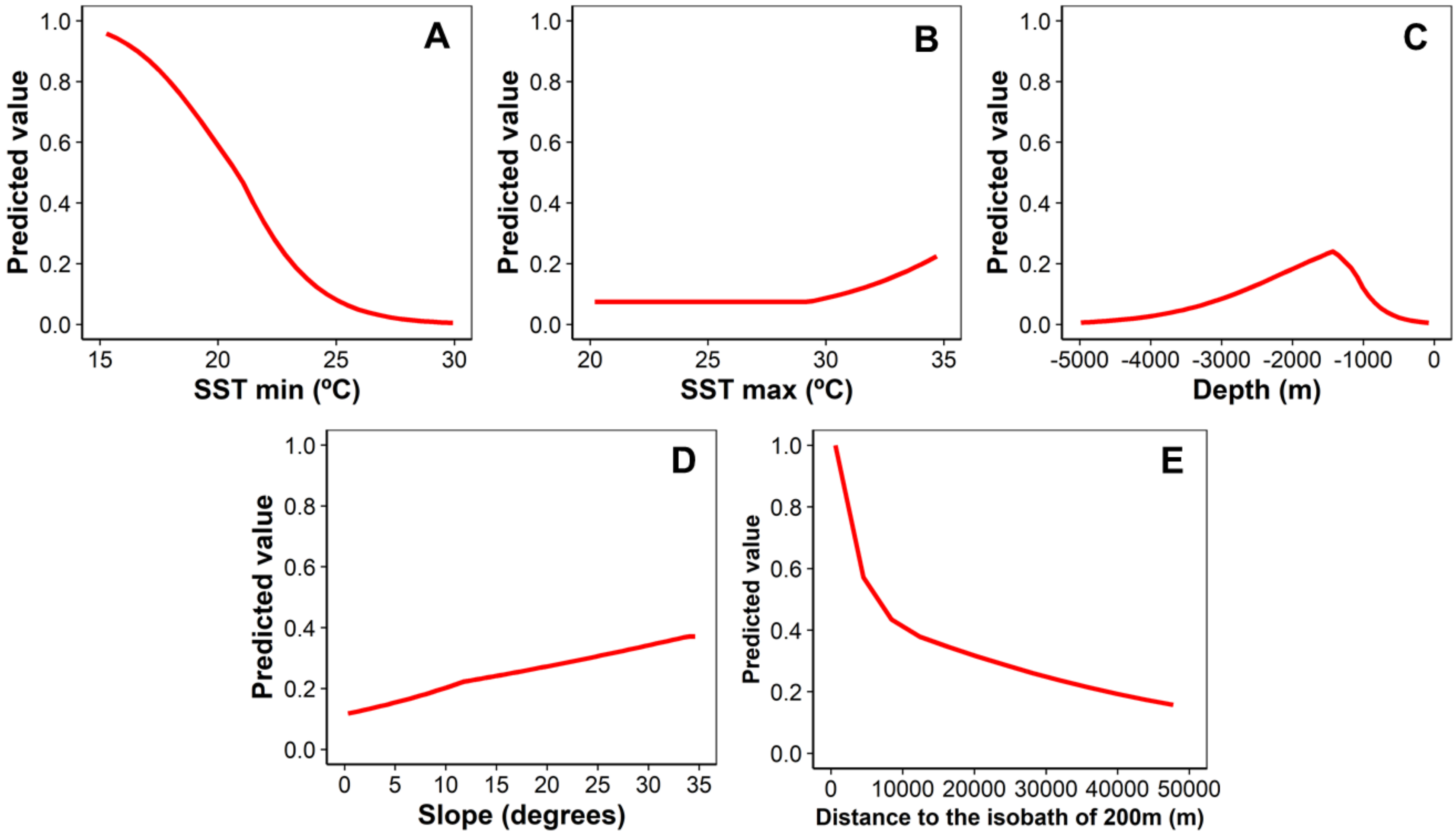

**Figure S4.** Short-finned pilot whale model. (A) $SST_{min}$, (B) Chl-$a_m$, (C) depth, (D) slope bottom, and (E) distance to the 200-m isobath.

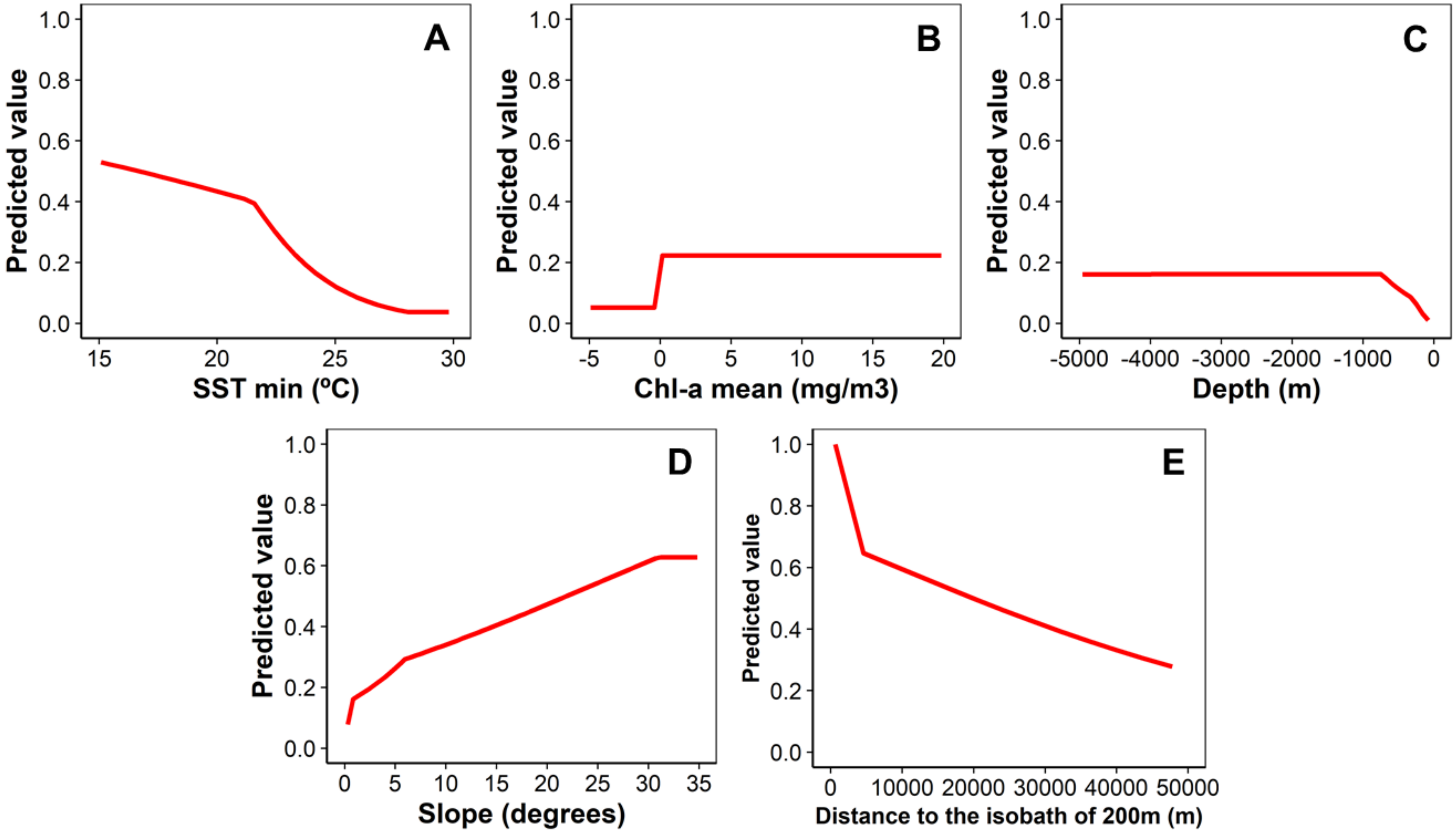

**Figure S5.** Rough-toothed dolphin model. (A) $SST_{min}$, (B) $SST_{max}$, (C) Chl-$a_m$, (D) slope bottom, and (E) distance to the 200-m isobath.

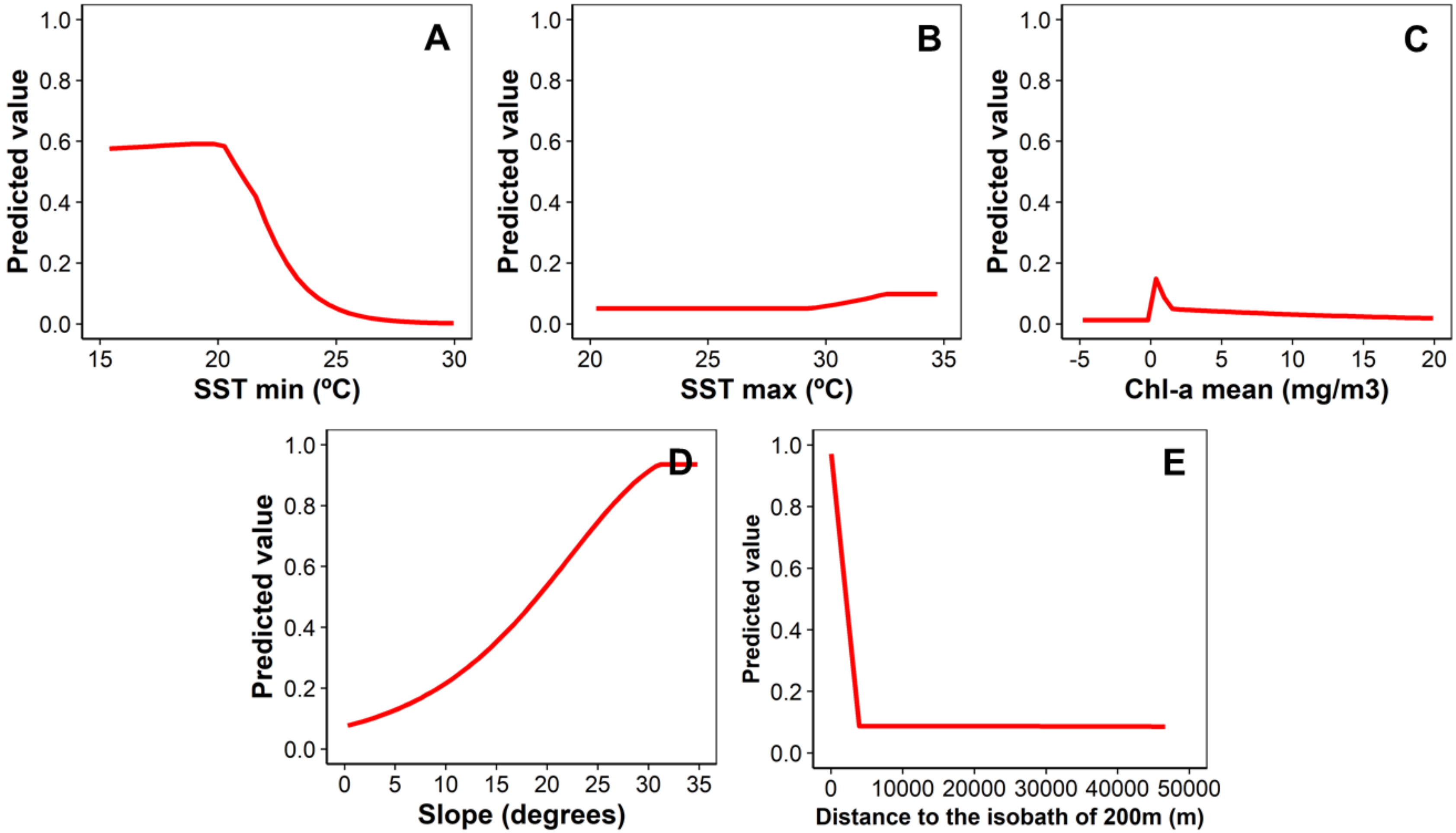

**Figure S6.** Risso's dolphin model. (A) $SST_{min}$, (B) $SST_{max}$, (C) Chl-$a_{m}$, (D) depth, and (E) slope bottom.

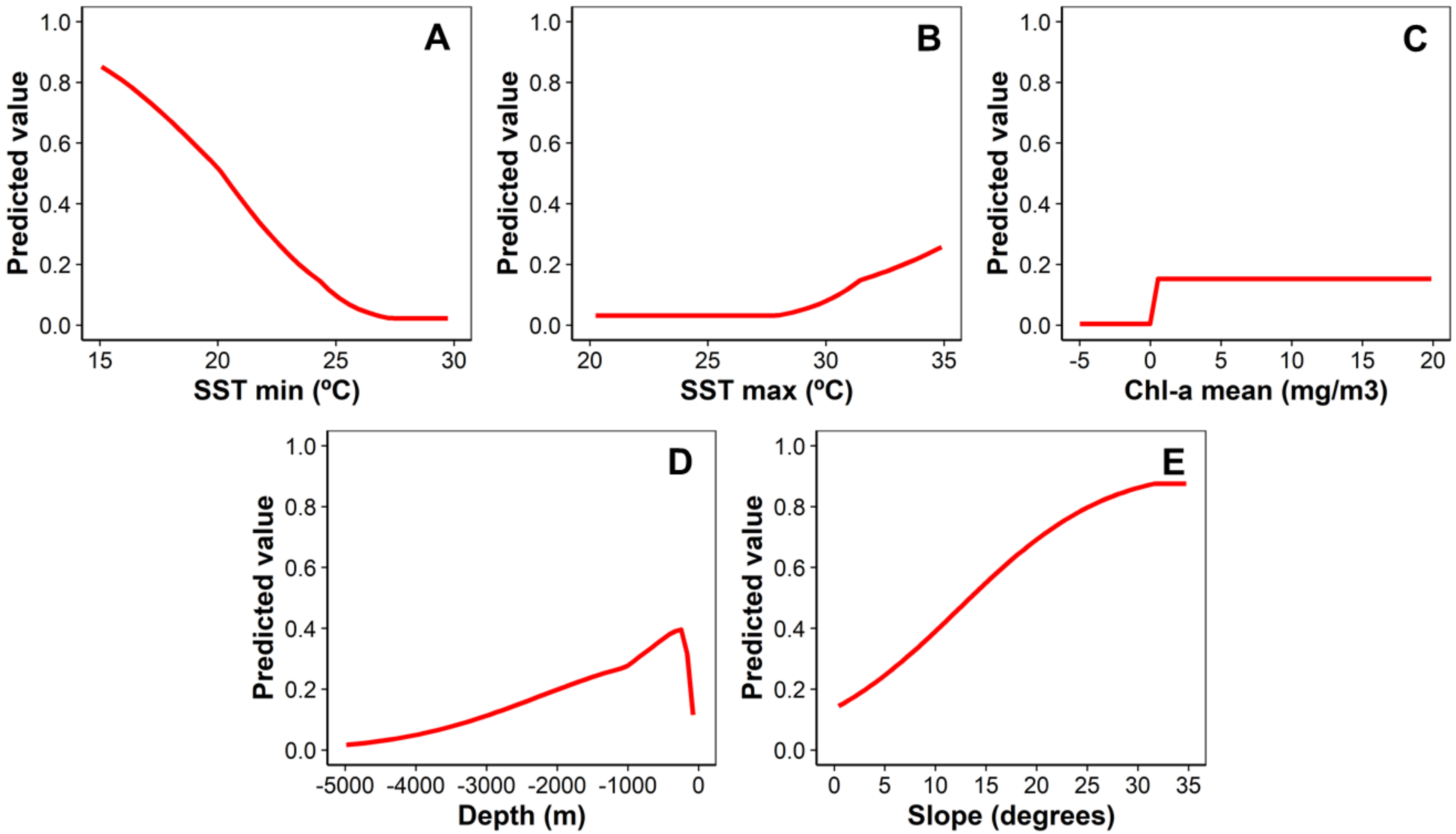

**Figure S7.** Atlantic spotted dolphin model. (A) $SST_{min}$, (B) Chl-$a_m$, (C) depth, (D) slope bottom, and (E) distance to the 200-m isobath.

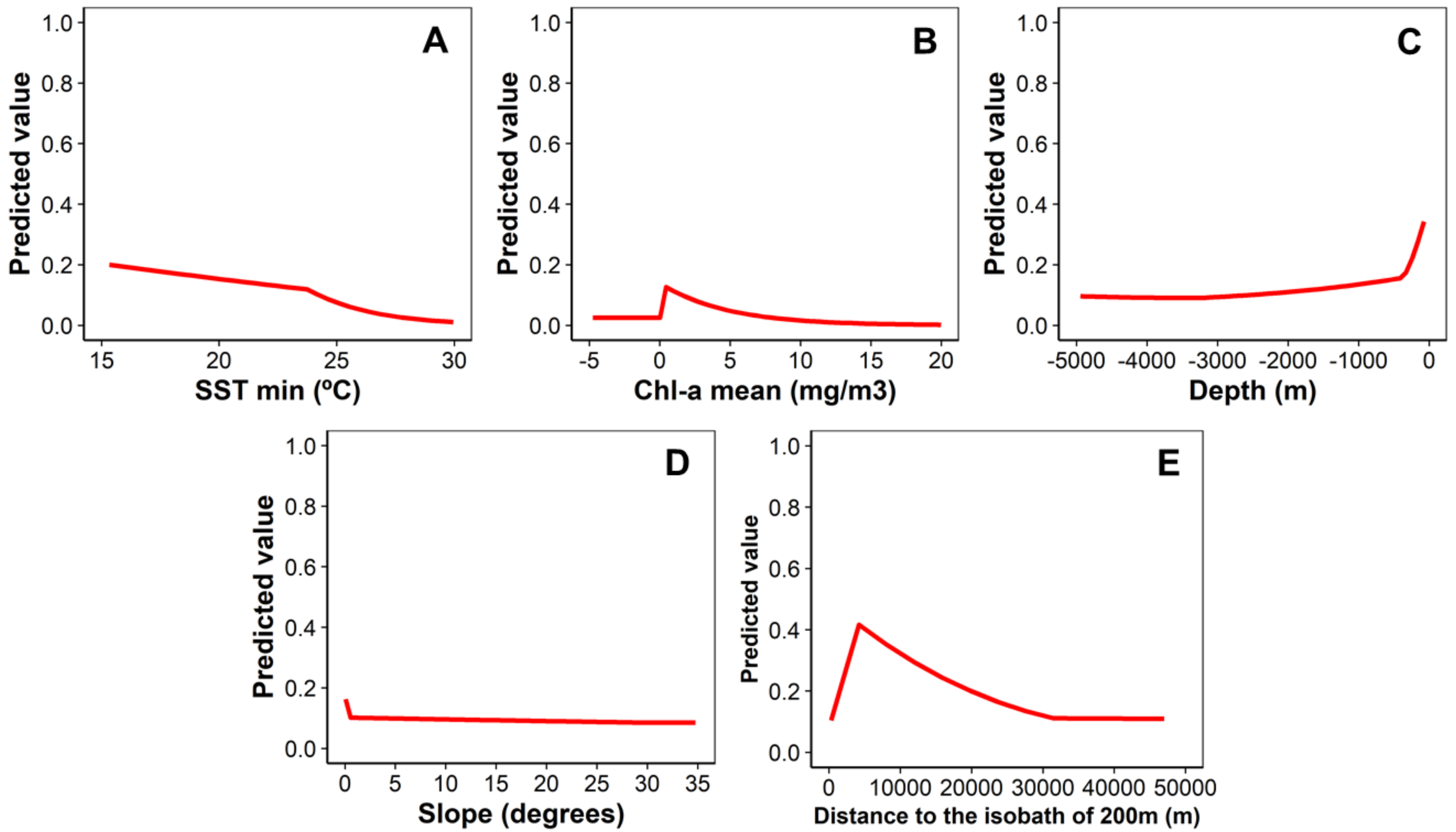

**Figure S8.** Pantropical spotted dolphin model. (A) $SST_{min}$, (B) $SST_{max}$, (C) Chl-$a_m$, (D) depth, (E) slope bottom, and (F) distance to the 200-m isobath.

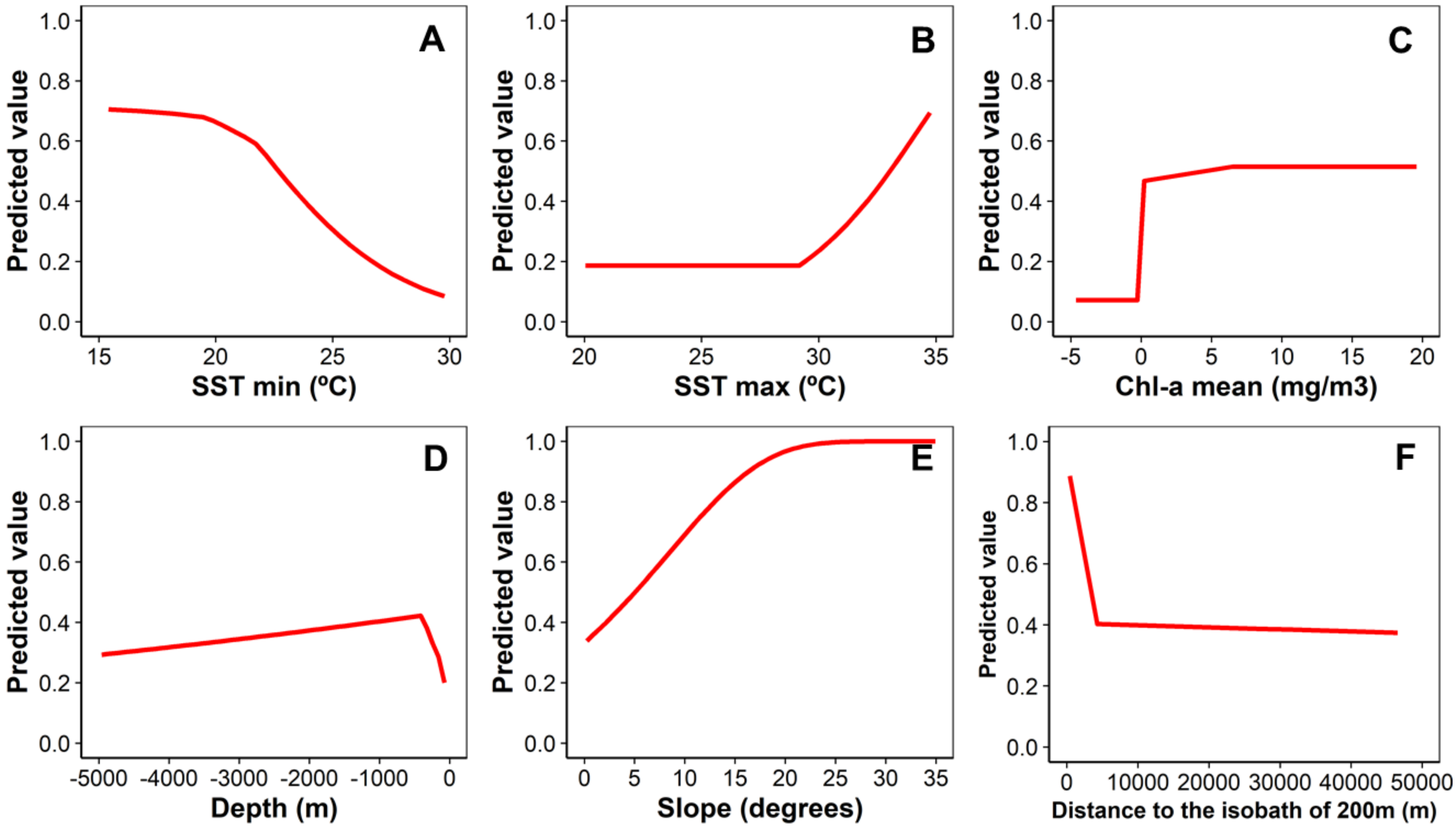

**Figure S9.** Striped dolphin model. (A) $SST_m$, (B) $SST_{max}$, (C) Chl-$a_m$, (D) depth, and (E) slope bottom.

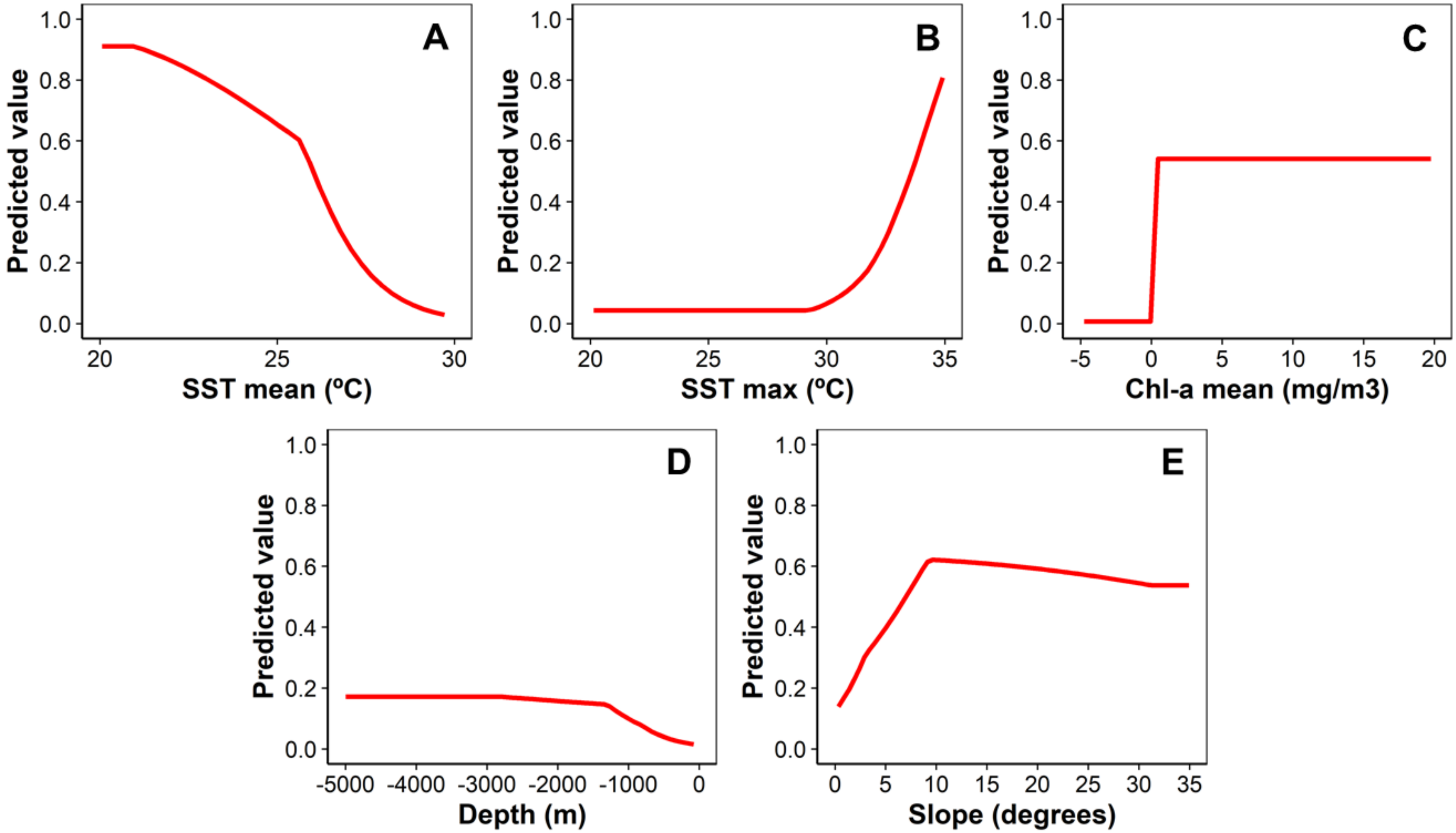

**Figure S10.** Spinner dolphin model. (A) $SST_{min}$, (B) $SST_{max}$, (C) Chl-$a_m$, (D) depth, (E) slope, and (F) distance to the 200-m isobath.

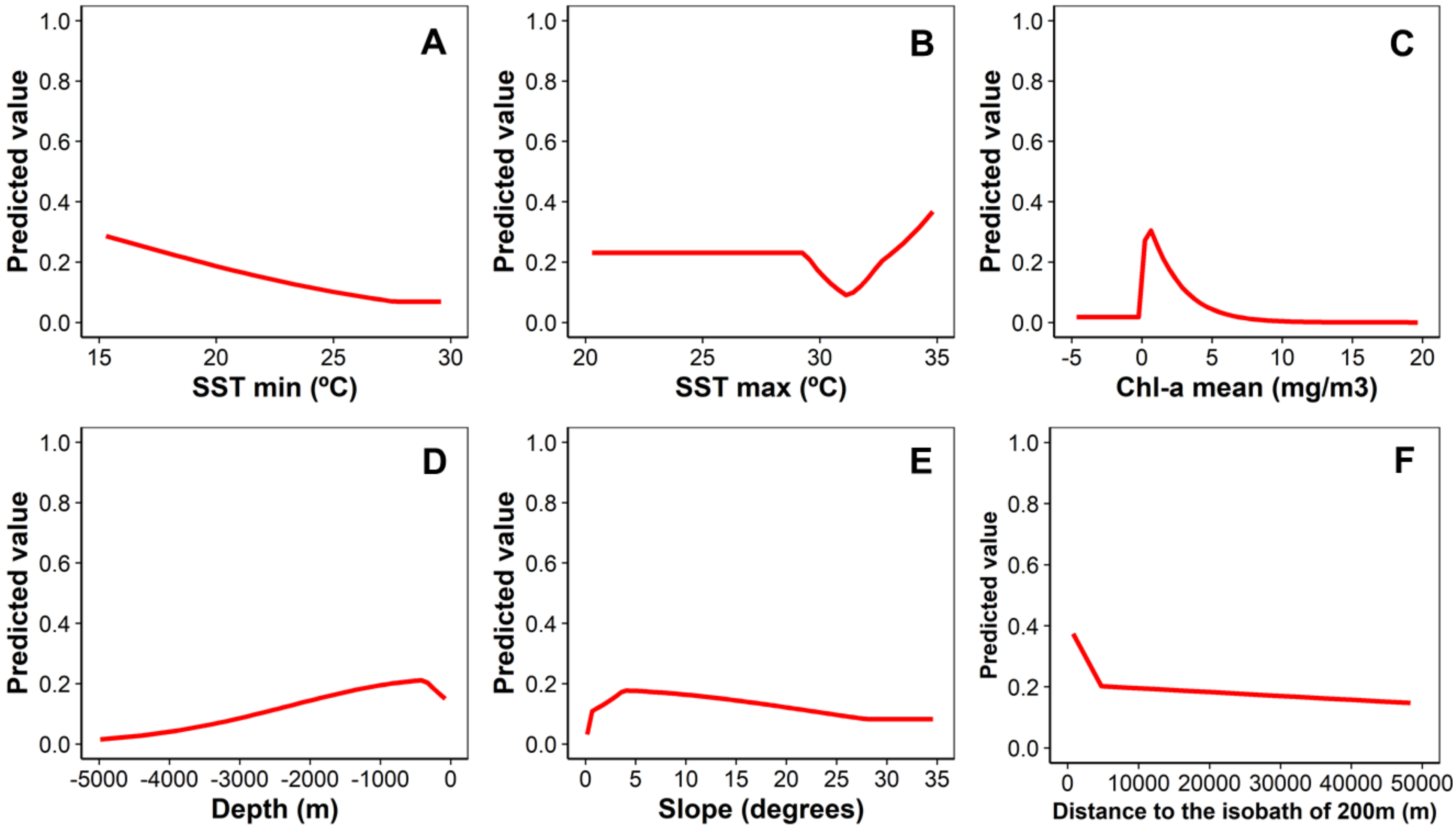

**Figure S11.** Clymene dolphin model. (A) $SST_m$, (B) $SST_{max}$, (C) Chl-$a_m$, (D) slope bottom, and (E) distance to the 200-m isobath.

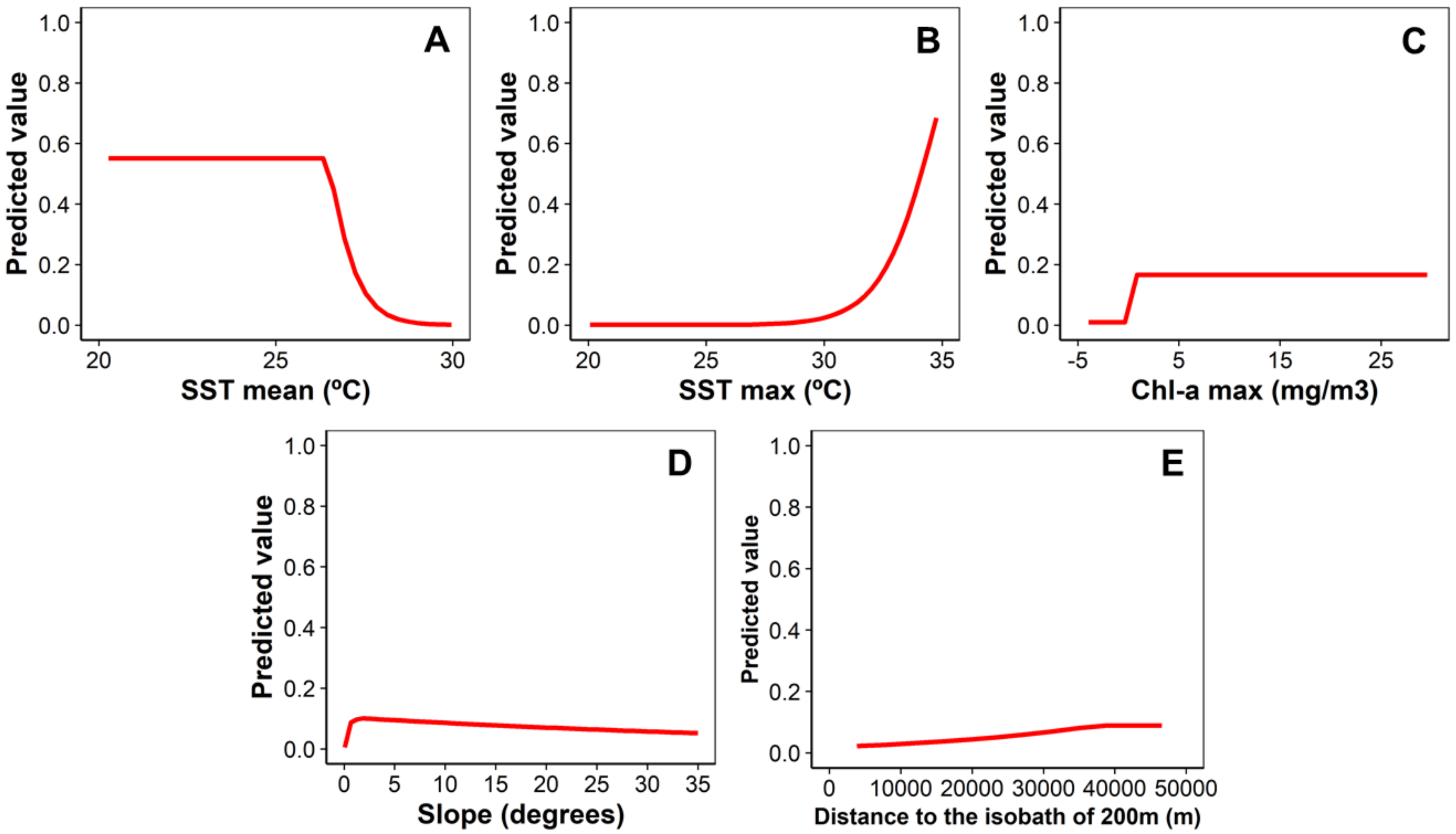

**Figure S12.** Bottlenose dolphin model. (A) $SST_{min}$, (B) $SST_{max}$, (C) Chl-$a_m$, and (D) depth.

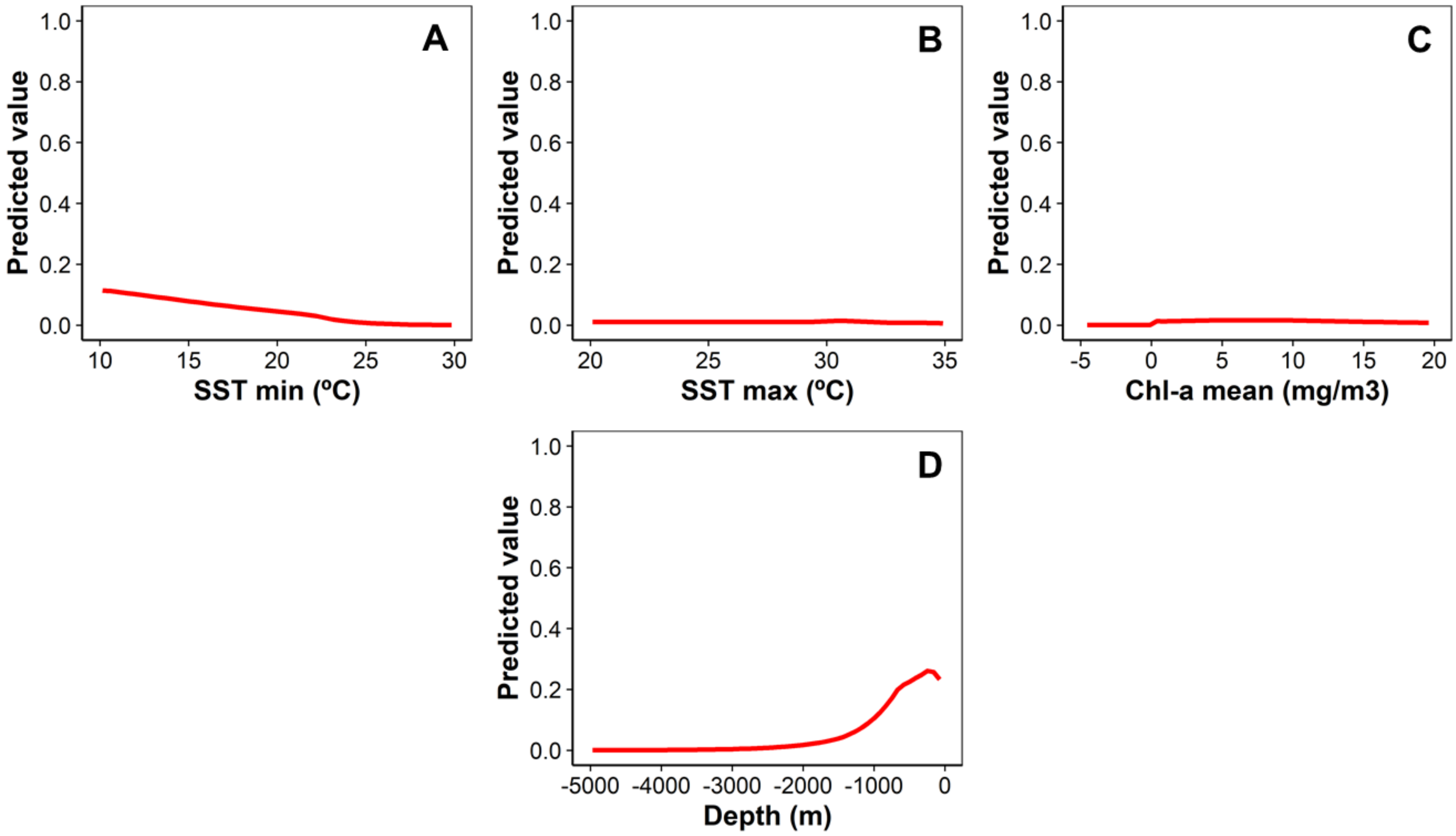

Supplement: Supplemental Information 4 — Figure S1. Sperm whale model. (A) SSTmin, (B) Chl-amax, (C) depth, (D) slope bottom, and (E) distance to the 200-m isobath. Figure S2. Dwarf sperm whale model. (A) SSTmin, (B) SSTmax, (C) Chl-amax, (D) depth, and (E) slope bottom. Figure S3. Cuvier’s beaked whale model. (A) SSTmin, (B) SSTmax, (C) depth, (D) slope bottom, and (E) distance to the 200-m isobath. Figure S4. Short-finned pilot whale model. (A) SSTmin, (B) Chl-am, (C) depth, (D) slope bottom, and (E) distance to the 200-m isobath. Figure S5. Rough-toothed dolphin model. (A) SSTmin, (B) SSTmax, (C) Chl-am, (D) slope bottom, and (E) distance to the 200-m isobath. Figure S6. Risso’s dolphin model. (A) SSTmin, (B) SSTmax, (C) Chl-am, (D) depth, and (E) slope bottom. Figure S7. Atlantic spotted dolphin model. (A) SSTmin, (B) Chl-am, (C) depth, (D) slope bottom, and (E) distance to the 200-m isobath. Figure S8. Pantropical spotted dolphin model. (A) SSTmin, (B) SSTmax, (C) Chl-am, (D) depth, (E) slope bottom, and (F) distance to the 200-m isobath. Figure S9. Striped dolphin model. (A) SSTm, (B) SSTmax, (C) Chl-am, (D) depth, and (E) slope bottom. Figure S10. Spinner dolphin model. (A) SSTmin, (B) SSTmax, (C) Chl-am, (D) depth, (E) slope, and (F) distance to the 200-m isobath. Figure S11. Clymene dolphin model. (A) SSTm, (B) SSTmax, (C) Chl-am, (D) slope bottom, and (E) distance to the 200-m isobath. Figure S12. Bottlenose dolphin model. (A) SSTmin, (B) SSTmax, (C) Chl-am, and (D) depth. [file peerj-09-10834-s004.pdf]
